# Predictive engines based on pharmacokinetics modelling for tacrolimus personalized dosage in paediatric renal transplant patients

**DOI:** 10.1038/s41598-020-64189-9

**Published:** 2020-05-05

**Authors:** Manuel Prado-Velasco, Alberto Borobia, Antonio Carcas-Sansuan

**Affiliations:** 1 Department of Graphics Engineering and Multiscale Modelling in Bioengineering Group, University of Seville, Seville, Spain; 2 La Paz University Hospital, School of Medicine, Autonomous University of Madrid, Madrid, Spain

**Keywords:** Computational models, Therapeutics, Kidney

## Abstract

The development of predictive engines based on pharmacokinetic-physiological mathematical models for personalised dosage recommendations is an immature field. Nevertheless, these models are extensively applied during the design of new drugs. This study presents new advances in this subject, through a stable population of patients who underwent kidney transplantation and were prescribed tacrolimus. We developed 2 new population pharmacokinetic models based on a compartmental approach, with one following the physiologically based pharmacokinetic approach and both including circadian modulation of absorption and clearance variables. One of the major findings was an improved predictive capability for both models thanks to the consideration of circadian rhythms, both in estimating the population and in Bayesian individual customisation. This outcome confirms a plausible mechanism suggested by other authors to explain circadian patterns of tacrolimus concentrations. We also discovered significant intrapatient variability in tacrolimus levels a week after the conversion from a fast-release (Prograf) to a sustained-release formulation (Advagraf) using adaptive optimisation techniques, despite high adherence and controlled conditions. We calculated the intrapatient variability through parametric intrapatient variations, which provides a method for quantifying the mechanisms involved. We present a first application for the analysis of bioavailability changes in formulation conversion. The 2 pharmacokinetic models have demonstrated their capability as predictive engines for personalised dosage recommendations, although the physiologically based pharmacokinetic model showed better predictive behaviour.

## Introduction

The immunosuppressant tacrolimus (TAC or FK506) is a calcineurin inhibitor (CNI) employed to reduce the risk of acute rejection and allograft loss of many types of solid organs. The clinical application of TAC has increased in recent decades, unlike other CNIs such as cyclosporine A, due to the lesser efficacy of the latter^1–3^. TAC is highly lipophilic and barely water soluble, which explains the high variability of its pharmacokinetic properties when orally administered^4^.

TAC is metabolised in liver microsomes mainly by CYP3A4 and CYP3A5 (in humans), to form a major metabolite with negligible pharmacological activity^4^. A second metabolite with equipotent activity to^4^ is scarcely produced.

The first commercial oral formulation of TAC was a fast-release hard gelatine capsule (Prograf), with a recommended 12 hours between administrations. The bioavailability of Prograf is approximately 20% in adults^5^; however, this is largely affected by numerous factors, including food intake. Advagraf, a prolonged-release formulation, was subsequently developed to facilitate treatment adherence with the once-daily administration of TAC (usually in the morning).

Selecting the optimal TAC dosage for patients who have undergone kidney transplantation (or other solid-organ transplantation) is difficult due to the narrow therapeutic index and large interpatient variability of TAC, the latter of which is associated with oral absorption variability, as well as the influence of CYP3A (cytochrome P450, family 3, subfamily A) and P-glycoprotein (PgP) enzyme activity and their polymorphisms^1^. TAC pharmacokinetics and toxicity are complex issues, and the need for individualised therapeutic drug monitoring (TDM) is well known^1^. Target TAC exposure recommendations maintain the trough blood TAC concentration (C0) as an alternative to blood TAC area under the curve (AUC), and various AUC ranges have been suggested as a function of the C0 target and formulation (twice daily for Prograf; once daily for Advagraf). The recommended C0 target for paediatric kidney transplant recipients at more than 2 months after transplantation is between 5 and 10 ng/ml, and the corresponding target ${{\rm{AUC}}}_{24}$ (AUC of blood TAC concentration during the last 24 h) is 180–350 ng/ml · h^6^.

The new TDM TAC consensus report includes for the first time the monitoring of intrapatient variability (IPV) exposure as a biomarker to predict treatment outcomes in kidney transplantation, although further research is required to validate the thresholds and the timing for measurements^6^. TAC IPV is defined as the TAC blood concentrations fluctuations within a subject over a period of time for which the TAC dose is constant^7^. Several mathematical formulations can be applied to compute the TAC IPV, in which the TAC blood concentrations are corrected for the drug dosage, D, when D is not stable^7,8^. Borra *et al*. found that a high TAC IPV was a risk factor for poor long-term outcomes in kidney transplantation^8^. Subsequent studies have confirmed the prevalence of large TAC IPV in the clinical setting and its influence on graft failure for long-term kidney transplant recipients^7,9,10^. The main factors that cause a high TAC IPV are considered to be the analytical assay, food, diarrhoea, drug-drug interactions (DDIs), genetic factors, nonadherence and the use of generic TAC formulations^7^.

Several pharmacokinetic TAC models have been developed to more accurately define dosages for target exposure, in line with the model-informed precision dosing (MIPD) approach^11^. The models include compartmental and noncompartmental approaches, as well as a physiological basis. We use the term “physiologically based pharmacokinetics” (PBPK) to refer both to individual and population-oriented models and employ the term PK to refer to the nonphysiological pharmacokinetic models.

The PK models for predicting TAC (Prograf formulation) concentrations in patients (with applications for dose adjustment) include a 1-compartment system with an intravenous (IV) drug administration model for paediatric haematopoietic stem cell transplant recipients^12^; several 2-compartment system models with first-order oral absorption for adult kidney transplant recipients^13^, adult liver transplant recipients^14^ and paediatric kidney transplant recipients^15^; and a 2-compartment system with absorption governed by a 3-compartment absorption and transit (CAT) subsystem for adult kidney transplant recipients^16^. Fewer studies with PK models have evaluated TAC concentration predictions with Advagraf, although the results from Benkali *et al*.^17^ for adult kidney transplant recipients showed that extended-release TAC can be assessed using a chain of transit regions for absorption. Outcomes from these cited studies have confirmed that PK models offer a suitable approach to achieve personalised TAC dosages, although the authors did not consider the patients’ temporal progression and real-time MIPD.

The PBPK approach has demonstrated better predictive capability than nonphysiologically based PK approaches^18^ and is therefore recommended when developing drug and clinical prescriptions for special populations, such as for paediatric patients^19^. PBPK models therefore seem more suitable than PK models for managing real-time MIPD^11^. However, most of the few studies about TAC distribution based on PBPK modelling are not designed for MIPD, but for other issues. These ones include the quantification of DDI mechanisms^20^ and the analysis of the sensitivity of TAC exposure to covariates^21^. This explains why those models had significant errors in TAC concentrations predictions, regardless of the use of an extensive whole-body approach^20^ or a simplified approach (reduced number of tissues)^21^. In addition, most of the published PBPK models were not designed to assess the patients’ progression.

To the best of our knowledge, there are no PK/PBPK models for TAC distribution that describe the modulation of TAC absorption and metabolism due to the circadian activity of CYP3A5 and PgP enzymes, despite the fact that the chronopharmacokinetics of TAC affect TAC exposure^22^ and is one of the causes of IPV^7^. Various human studies have shown that the maximum blood concentration (C_max_) and AUC (of blood concentration) after the morning dose are higher than the values after the evening dose^23^, although there are questions regarding the mechanisms involved.

Real-time MIPD in TAC prescription provides a customised TAC dosage (output), calculated as a function of previous TAC measurements and other clinical and physiological data (inputs), which presents a real-time optimisation problem that requires a mathematical model able to predict the dynamics of TAC concentration (and other variables) in the desired tissues. The suitability of real-time MIPD is associated with the predictive capability of the mathematical model, which in turn requires the use of adaptive techniques to correct intrapatient variability^24^. A well-known application of real-time MIPD is glycaemic control through insulin therapy, particularly in intensive care units^25^. However, the use of real-time MIPD in TAC requires further research.

The goal of this study is to develop 2 TAC models based on the PK and PBPK methodologies and to evaluate and compare the models as predictive engines for real-time MIPD. The PK model is based on the model created by Andreu *et al*.^16^, whereas the new PBPK model uses the model created by Gerard *et al*. as a reference^21^. The specific objectives of this study are (i) to explore the goodness of fit and predictive capability of both models for twice-daily Prograf administration after the inclusion of the chronopharmacokinetics of TAC, testing the TAC circadian mechanistic proposal of Baraldo *et al*.^23^ and the improvement in predictive accuracy of PBPK versus PK models^18^; (ii) to evaluate the capability of several models’ adaptive techniques based on prior parameter distribution to analyse the conversion from Prograf to Advagraf formulation and to calculate intrapatient variability through the parametric mechanisms involved; and (iii) to analyse the change in absolute bioavailability during formulation conversion according to the models’ predictions.

## Methods

The study was performed in 2 phases. During the first phase, we developed, fitted and evaluated population PK and PBPK TAC models using a retrospective stable paediatric population with kidney transplants who were administered Prograf twice daily (Prograf data). During the second phase, we examined the new models’ capability to attend the patients’ progression and evaluated several personalised optimisation strategies to adapt the models to the same paediatric patient group, after transitioning from the Prograf to the Advagraf formulation (Advagraf data). We employed adaptive techniques (such as Bayesian estimation)^26^ based on the models’ parameter distributions obtained during the first phase to adjust the models to the patients’ progression. This strategy explains why our design uses population PK models instead of individually based models, which are limited to more typical adaptive techniques from the adaptive control theory^24^ applied in pharmacological controls.

The population PK and PBPK mathematical models and adaptive techniques are presented in the next 3 subsections, and the computational procedures for the study’s 2 phases are presented in the last subsection (Model Analysis). A glossary with the names of the variables, definitions and units is presented for each mathematical model. Additional details and data regarding the models can be found in the First Supplementary Document.

The mathematical models were implemented on multilevel object-oriented PhysPK biosimulation software. An evaluation of the accuracy and underlying methodology of PhysPK with respect to NONMEM software was recently published^27^. The model fitting, covariate analysis, statistics and optimisation procedures described in the next sections are also implemented in PhysPK. We successfully compared the PhysPK population-fitting techniques against NONMEM^28^. Further details regarding these computational procedures appear in the First Supplementary Document.

### Pharmacokinetic model development

A 2-compartment structural model with a 3-compartment absorption and transit (CAT3) subsystem was the basis of the PK model for TAC distribution. The CAT3 subsystem is based on the model for oral drug absorption defined in the study by Lawrence *et al*.^29^, in which the small intestine is addressed as a series of 3 segments, and absorption in the stomach and colon is ignored^30^.

Similar PK models with more compartments were tested without improvement in either TAC prediction or IPV. The model’s mathematical equations are detailed in the First Supplementary Document, while Table 1 shows the model variables. TAC elimination was assumed to be linear and given by the central clearance $Cl$. TAC transit in CAT3 is assumed to be linear, with a mean transit time in the stomach, small intestine and colon equal to $MTT$. The transit rate constant is ${k}_{t}=1/MTT$.

**Table 1:** Definition of the Variables employed in the TAC PK model.

Variable	Units	Description
$Cl$	L/h	Systemic clearance
$C{l}_{d}$	L/h	Distribution clearance
${V}_{i}$	L	Volume of compartment i^†^
${C}_{i}$	ng/ml	Concentration at compartment i^†^
${K}_{a}$	ml/h	Absorption constant
$MTT$	h	Mean transit time
${r}_{chr}$	pu	Chrono modulation factor
${t}_{chr}$	h	Chrono modulation temporal phase
$D$	mg	Dose
${k}_{t}$	1/h	Transit rate constant
${V}_{cat,j}$	L	Fluid volume of zone j into CAT^‡^
${k}_{ta}$	1/h	Absorption rate constant

^†^i takes values c (central) or p (peripheral). ^‡^j takes values s (stomach), si (small intestine), and c (colon).

**Figure 1:**
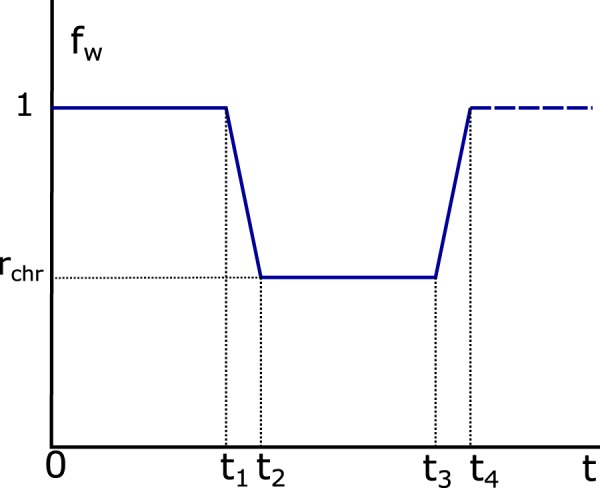
24-h periodic waveform ${f}_{w}(t)$ (pu) with ${t}_{1}=10,{t}_{2}=12,{t}_{3}=22$ and ${t}_{4}=24\,{\rm{h}}$.

The systemic clearance $Cl$ and absorption constant ${K}_{a}$ were modulated with a circadian profile according to the suggestions from the Baraldo and Furlanut study^23^, based on experimental and clinical studies. Chronomodulated parameters were calculated using Eq. (1).

1 $$\begin{array}{rcl}C{l}_{chr}(t) & = & Cl\cdot {f}_{w}(t-{t}_{chr})\\ {K}_{achr}(t) & = & {K}_{a}\cdot {f}_{w}(t-{t}_{chr}),\end{array}$$

in which $${f}_{w}\,(t)$$ is a 24-h periodic waveform function (Fig. 1). As shown in Fig. 1, the chronomodulated clearance ($$C{l}_{chr}$$) is kept equal to $$Cl$$ during the diurnal phase of the cycle (0 − $${t}_{1}$$) and reduced with the factor $${r}_{chr}\le 1$$ during the nocturnal phase ($${t}_{2}-{t}_{3}$$). Diurnal-nocturnal ($${t}_{1}-{t}_{2})$$ and nocturnal-diurnal ($${t}_{3}-{t}_{4}$$) phases are completed in 2 h with a constant slope ramp. The value $$time=0$$ is defined as the reference time for the first TAC measurement in the clinical study (Methods section). The diurnal phase could therefore start at a different time. This is modelled with the waveform function $${f}_{w}(t-{t}_{chr})$$, in which the diurnal phase starts at $$t={t}_{chr}$$. The reduction factor $${r}_{chr}$$ and the diurnal starting time reference $${t}_{chr}$$ are variable population parameters. The defined circadian profile is the simplest function that supports the circadian behaviour of TAC $$Cl$$ according to current knowledge. $${K}_{achr}$$ was defined with the same function $${f}_{w}$$.

The absolute drug bioavailability ($F$) was calculated using Eq. (2):

2 $$F(t)\cdot {\int }_{0}^{t}\,{D}_{f}(t)\cdot {\rm{d}}t={\int }_{0}^{t}\,C{l}_{chr}(t){C}_{c}(t)\cdot {\rm{d}}t$$

in which ${D}_{f}$ is the drug mass flow rate. The TAC dosage ($D$) was determined with Eq. (3).

3 $$D={\int }_{0}^{FDT}\,{D}_{f}\cdot {\rm{d}}t$$

Equations (2) and (3) were calculated during the simulation to provide $F(t)$ bioavailability.

### Physiologically based pharmacokinetic model development

**Figure 2:**
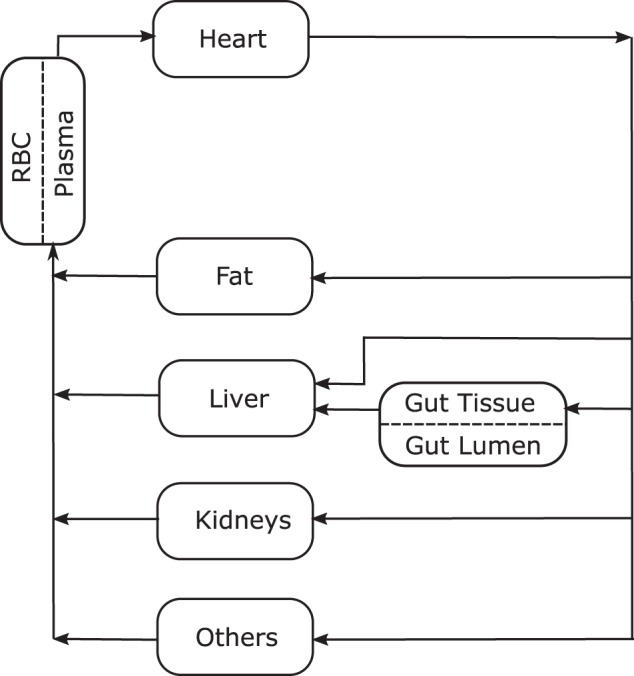
Flow diagram for TAC PBPK model with 4 flow-limited tissues (fat, kidneys, liver and others) and 2 membrane-limited tissues (gut and blood). The blood compartment is defined through the red blood cell-plasma component. The gastric system is comprised of a gut lumen where the TAC form is liberated following a zero-order kinetic with sink condition, a one-order absorption membrane and gut tissue perfused with blood.

The PBPK TAC model comprises gastric, liver, kidney and primary blood tissues, together with fat tissue and other tissue. The input variables, such as body weight (BW), sex, age, haematocrit and CYP3A4/5 activity, were considered using mechanistic mathematical equations, which are described in this section. The flow diagram is shown in Fig. 2.

All tissues are nonhomogeneous for TAC, with tissue-plasma partition coefficients associated with the lipophilic character of this drug. The partition coefficients were calculated using the Poulin and Theil method, based on the solubility of drugs in neutral lipids, phospholipids and water^31^. Physiological lipid and water fractions in tissues were taken from published databases^31–33^.

The model’s mathematical equations follow usual PBPK assumptions. However, they are written in detail in the First Supplementary Document. Table 2 shows the variables used in the model.

**Table 2:** Variables employed in the TAC PBPK model.

Variable	Units	Description
${V}_{i}$	ml	Volume of tissue i^†^
${C}_{i}$	ng/ml	i tissue concentration^†^
${C}_{vi}$	ng/ml	Venous i tissue concentration^†^
${C}_{ut}$	ng/ml	Unbound t tissue concentration^‡^
${C}_{bt}$	ng/ml	Bound t tissue concentration^‡^
${R}_{t}$	pu	Tissue (t) - plasma partition ratio^‡^
${Q}_{i}$	ml/min	Venous blood perfusion flow rate in i tissue^†^
$f{u}_{p}$	pu	Unbound plasma fraction
$f{u}_{t}$	pu	Unbound tissue fraction^‡^
${B}_{max}$	$\mu$ g/L	Maximum binding capacity in red blood cells
${K}_{d}$	$\mu$ g/L	Dissociation constant in red blood cells
$C{l}_{ih}$	1/h	Intrinsic hepatic clearance per liver volume
${K}_{abs}$	1/h	Absorption rate constant
$LibT$	min	TAC liberation time
${K}_{elib}$	1/h	Elimination rate constant in liberation region
${r}_{chr}$	pu	Chrono modulation factor
${t}_{chr}$	h	Chrono modulation temporal phase
$D$	mg	Dose

^†^i takes values l (liver), k (kidney), g (gut tissue), f (fat), o (others), b (blood). ^‡^t takes the same values as i, except b.

TAC plasma binding is defined as the unbound plasma fraction $f{u}_{p}$, which was 0.012^34^, despite some variability. This value is commonly used in other modelling studies^21,35^. An exploratory Monte Carlo simulation was performed to confirm the model’s robustness. Details are presented in the First Supplementary Document.

TAC demonstrated extensive binding to red blood cells, with a maximum binding capacity ${B}_{max}$ of 418 ± 258 *μ*g/L and dissociation constant (${K}_{d}$) of $3.8\pm 4.7$ *μ*g/l for adult liver transplant recipients^36^. Due to the importance of TAC binding to red blood cells, its variability with different populations (e.g., healthy individuals have approximately double the value of ${B}_{max}$^36^) and the lack of data for paediatric renal transplant recipients, these parameters have been set as population variables to be fitted.

We assumed that TAC elimination is due only to liver metabolism and that renal excretion is negligible^2,21^. We calculated the total intrinsic hepatic clearance as $C{l}_{ih}\cdot {V}_{l}$ (ml/h), with ${V}_{l}$ the liver volume (ml).

Liberation is governed by the zero-order rate liberation constant ($\mathrm{1/}LibT$) in which $LibT$ is the TAC form liberation time (min). Gut absorption is defined by the one-order absorption rate constant ${K}_{abs}$ (1/h), with a value of 4.5 1/h^36^.

The one-order elimination rate constant ${K}_{elib}$ (1/h) describes the TAC form that cannot be absorbed from the gut lumen, which is related to $LibT$ through Eq. (4), which states that elimination increases with $LibT$. The relationship is based on the assumption that higher LibT induces the transit of a larger portion of unabsorbed TAC through the intestine.

4 $${K}_{elib}(LibT)={K}_{elib}(LibT=100)\cdot \frac{LibT}{100}$$

Model parametric fitting in the exploration phase provided better TAC predictions for ${K}_{elib}$ defined by Eq. (4) than with the constant ${K}_{elib}$ and other polynomial ${K}_{elib}-LibT$ relationships. We assumed that $LibT$ depends mainly on the release time of the TAC formulation, which is shown in the additional model data section of the First Supplementary Document.

Intrinsic hepatic clearance ($C{l}_{ih}$) and the gut absorption rate constant (${K}_{abs}$) were modulated with a circadian profile similar to that employed in the PK model. The chronomodulated values $C{l}_{ihchr}(t)$ and ${K}_{abschr}(t)$ are defined by Eq. (1), replacing $Cl$ and ${K}_{a}$ with $C{l}_{ih}$ and ${K}_{abs}$, respectively. The bioavailability $F$ was computed using Eq. (2), replacing $C{l}_{chr}(t)\cdot {C}_{c}$ with ${V}_{l}\cdot C{l}_{ihchr}(t)\cdot {C}_{b}$.

### Adaptive techniques

We fitted models to our study patients using a maximising population likelihood approach followed by customisation for each patient (Bayesian optimisation^26^). We then employed adaptive techniques to adjust the models’ parameters to deviations associated with patient temporal intravariations and to modifications in the external context or therapies (such as changes in drug formulations). The adaptive techniques evaluated in this study are as follows:

1. Adaptive Bayesian optimisation.
2. Adaptive weighted least squares (WLS) optimisation.

The clinical protocol is described below. Both techniques benefit from the fact that the target patient belongs to a known prior population, which explains why central (mean) parameters remained constant during the customised adaptation phase.

The adaptive Bayesian technique follows the same equations of the well-known Bayesian method^26^, with the exception of boundary conditions. If $\eta$ is the interpatient variation of the population variable $\phi$ (see Model analysis Section) to be computed, this variable is restricted to fulfilling one of the two following boundary conditions (Eq. (5)):

5 $$\begin{array}{rcl}-\,{W}_{b}\cdot \,{\rm{\min }}({\eta }_{0},{\eta }_{m}) & \le  & \Delta \eta \le {W}_{b}\cdot \,{\rm{\min }}({\eta }_{0},{\eta }_{m})\\ -\,B & \le  & \Delta \eta \le B,\end{array}$$

in which ${\eta }_{0}$ is the value of $\eta$ at the previous model state (time ${t}_{0}$), Δ$\eta =\eta -{\eta }_{0}$ is the increment of $\eta$ in the readjustment and ${\eta }_{m}$ is the minimal $\eta$ value that defines the weighted boundary. ${W}_{b}$ is the boundary Weight and $B$ is the absolute Boundary. Both equations limit the variation of $\eta$ with respect to the previous state to a weighted value of ${\eta }_{0}$ or to an absolute boundary B. Selection of a B-based or W-based boundary is based on the knowledge concerning the associated population variable $\phi$.

The adaptive WSE optimisation minimises the objective function ${F}_{wse}$ given by Eq. (6):

6 $${F}_{{\rm{wse}}}=W\,\sum _{{t}_{k}}\,{(\mathrm{ln}({C}_{b}({t}_{k}))-\mathrm{ln}(\widehat{{C}_{b}}({t}_{k})))}^{2},$$

in which $W$ is the weight applied to the squared difference between blood model tacrolimus concentration ${C}_{b}({t}_{k})$ and blood measured tacrolimus concentration $\widehat{{C}_{b}}({t}_{k})$, and ${t}_{k},k=1,2,\ldots n$ are the sample times.

As opposed to the adaptive Bayesian technique, which can only be applied to adjust parameters pertaining to the prior population set, adaptive WLS optimisation can also be used to adjust other models’ variables that were considered constant in the initial design, which helps extend the model’s scope. Adjusted variables and their boundary equations are fully detailed in the Adaptive Modelling Techniques section in the First Supplementary Document.

### Clinical study

The clinical study was designed as a single-centre, open-label TAC treatment conversion from a Prograf to Advagraf formulation (1:1, mg:mg), which included stable paediatric kidney transplant recipients. It was conducted at La Paz University Hospital (referral hospital for paediatric kidney transplantation in Madrid, Spain). The study protocol was approved by the Ethics Committee of La Paz University Hospital and the Spanish Agency for Medicines and Health Products. The study was registered under European Clinical Trial Register EudraCT 2009-017600-89. All methods were performed in accordance with required guidelines and regulations. Written informed consent was obtained prior to the patients’ enrolment from their parents or guardians, who controlled the patient’s drug administration and food intake according to the instructions provided. A full detailed description of the clinical study design, including the results of the evaluation of the relative bioavailability of Prograf and Advagraf formulations, has been published^37^. Data collected during the pharmacokinetic phase of the study (2 weeks), which is described in the following paragraphs, support the development of the present research with the aim of reaching the 3 specific objectives stated in the Introduction Section.

Patients with a stable renal transplant and younger than 18 years of age were recruited according to the inclusion criteria. The study size was defined as 21 patients, although only 18 patients were needed to achieve statistically significant steady-state ${{\rm{AUC}}}_{24}$ (AUC of blood TAC concentration during the last 24 h) target differences of 5% with a power of 80%^37^. The inclusion criteria included a variation in TAC dosage and concentrations lower than 20% during the last 30 days prior to the start of the study, without administering drugs that might affect the TAC PK in the last 15 days. None of the patients dropped out during the pharmacokinetic study period, and none of the patients experienced adverse events during the 2 weeks of the PK study. None of the concomitant treatments were modified during the study.

The mean time from transplant was $5.39\pm 3.25$ years, and nearly all patients were white (17). The remaining patients were Hispanic (1), Asian (2) and Arabian (1). The sex distribution was 57% male/43% female. The other anthropometric data (expressed as mean ± SD, range) were age (12.9 ± 4.17 years, 4–17), body weight (42.85 ± 15.42 kg, 15.1–63.8) and height (143.4 ± 18.16 cm, 105–168).

The patients were administered a controlled Prograf regimen the week prior to their first admission to La Paz University Hospital (day 1). The patients remained on Prograf during day 1, which was first administered between 8:00 AM and 9:00 AM and then re-administered 12 h later. The drug was administered with 200 ml of water, and the patients ate breakfast, lunch and dinner 11:00 AM, 1:00 PM and 7:00 PM. During the hospital stay, blood TAC was measured just before the morning TAC administration ($t=0$) and consecutively 0.5, 1, 1.5, 2, 3, 4, 6, 8, 12, 12.5, 13, 14, 15, 16, 18, 20 and 24 h later (a total of 18 measurements). Blood samples (2 ml) were collected in aliquot duplicate Ethylene Diamine Tetraacetic Acid (EDTA) tubes and analysed with an enzyme immunoassay (Dimension, Siemens Healthcare Diagnostics, Frimley, Camberley, UK), with 2 ng/ml as the lower quantification limit and 30 ng/ml as the upper limit. Measurements above the upper limit were diluted to obtain concentrations in the valid range.

Prograf was replaced with Advagraf on the following morning (day 2) with a new controlled protocol. Once-daily Advagraf doses were based on 1:1 total dose equivalence. Patient data acquired during the Prograf phase of the study (day 1) were used to fit and evaluate the new population PK and PBPK TAC models. The patients were re-admitted to La Paz University Hospital on day 8, repeating the food intake and blood sampling procedures of the first monitoring day, with the following sampling times: 0, 0.5, 1, 1.5, 2, 2.5, 3, 4, 6, 8, 12, 15 and 24 h (13 measurements). The blood TAC measurement ($t=0$) of this second series was performed just before administering Advagraf. The sample was the trough blood TAC concentration (C0) at the beginning of the hospital re-admission day. The model’s parameters were readjusted to reduce blood TAC concentration prediction errors during day 8. The adaptive optimisation techniques for the model readjustment are presented in the Adaptive techniques Section. The TAC measurements and data used for the analysis are included in the Second Supplementary File.

### Models analysis

#### Population model structure

The population parameters with interindividual variability in the PK model were $Cl$, ${K}_{a}$, $MTT$, ${r}_{chr}$ and ${t}_{chr}$ (variable parameters). The population PK parameters without interindividual variability were ${V}_{c}$, ${V}_{p}$ and $C{l}_{d}$ (nonvariable parameters). This set of parameters agrees with those selected by Andreu *et al*.^16^, except for new circadian parameters. Another nonvariable parameter was the coefficient of the final accepted covariate model, which is presented below.

All population parameters in the PBPK model have interindividual variability and included $C{l}_{ih}$, ${B}_{max}$, and ${K}_{d}$. The variables ${r}_{chr}$ and ${t}_{chr}$ were added for circadian modulation as in the PK model.

The interindividual error model for variable population parameters was exponential, assuming log-normal distribution for error ($\eta$), with a mean value $\theta$, as shown in Eq. (7) for the $Cl$ parameter.

7 $$Cl={\theta }_{Cl}\cdot {e}^{{\eta }_{Cl}}$$

We then defined the normal distribution for the $\eta$ vector (dimension equal to the number of variable population parameters) using the zero mean and covariance matrix Ω. We employed the log-transformed whole blood TAC concentration (ln *C*_*c*_ in PK and ln *C*_*b*_ in PBPK) as the model output to be fitted. We tested the proportional and additive residual error to establish the model that best fits the clinical data.

Interoccasion variability (IOV) reflects random IPV that may be transferred to the residual variability term if it is not considered^38^. However, the term IOV is associated with occasions defined by dosing periods, number of samples, or administration instants, which must be anticipated to be properly implemented. This is not the case of a model that may be applied as a customized knowledge engine in a real-time MIPD for different clinical environments and changing drug administration scheduling. Therefore, we decided not to implement IOV in the models structure.

#### Covariates analysis

The variables BW, body mass index, body surface area (BSA, calculated from BW and height using the Du Bois formula), sex, age, height and haemoglobin concentration were tested as potential covariates with an influence on PK model parameters, using a forward stepwise approach^39^. The PBPK model included available covariates by means of equations describing mechanistic models, as described in the previous section.

We analysed the correlations during the initial exploration, and the correlation models were linear or exponential for continuous covariates. The linear model correlation is as follows:

8 $${\theta }_{i}={\hat{\theta }}_{i}\cdot (1+{\theta }_{c}\cdot (x-{x}_{r}))$$

whereas the exponential model is as follows:

9 $${\theta }_{i}={\hat{\theta }}_{i}\cdot {\left(\frac{x}{{x}_{r}}\right)}^{{\theta }_{c}},$$

in which ${\hat{\theta }}_{i}$ is the mean population variable without covariate influence, ${\theta }_{c}$ is the correlation coefficient, $x$ is the covariate and ${x}_{r}$ is the reference value for this covariate. The last value was taken as the median value. The value of ${\theta }_{c}$ was estimated during the fitting population process. Categorical covariates such as sex were analysed assigning discrete values to each categorical level of the covariate.

The best correlation models were selected to be evaluated during the forward stepwise approach. We accepted a covariate-population variable relationship if the reduction in the minimum value of the objective function (MVOF) was somewhat less than 3.84 (significance level of 5% according to ${\chi }^{2}$ distribution for 1 degree of freedom in the nested model). In addition, an approximate reduction of 10% in intraindividual variability (coefficient of variation [CV]) was required in the associated population parameter for minimum clinical significance.

#### Population model fitting techniques and evaluation

We performed the population model fitting with the blood TAC concentrations measured during the first monitoring day in the hospital (Prograf administration), executed in 2 steps. During the first step, we also performed an initial exploration of the models to ensure their robustness and reliability.

We estimated the population parameters as a second step by maximising the likelihood function associated with the hierarchical models, using first-order conditional estimation methods (FOCE and FOCE-I)^40,41^. The absolute simulation time at the first hospital admission was set at 48 h. Additional details can be found in the Model analysis – Population fitting section in the First Supplementary Document.

The initial evaluation of the models’ behaviour was based on individual residual errors, conditional weighted residual, $\varepsilon$-shrinkage, $\eta$-shrinkage^42^ and relative standard errors (RSEs) of estimated parameters.

We also performed a noncompartmental analysis (NCA) based on TAC blood concentration measurements to compute the NCA ${{\rm{AUC}}}_{24}$ (log-trapezoidal method), differences model ${{\rm{AUC}}}_{24}$ minus the NCA ${{\rm{AUC}}}_{24}$ (Δ_AUC_) and the root mean square error (RMSE)^43^ of the blood TAC concentrations minus the model TAC concentrations (log transformed) ${{\rm{RMSE}}}_{c}$.

We performed goodness of model predictions by plotting the predictive simulated TAC concentrations against the measured blood TAC concentrations, showing the accuracy and characteristic dynamics of the computed TAC concentrations. We performed the prediction-corrected visual predictive check (pcVPC)^44^ with 500 simulated replicas of the patients’ data set to confirm the proper adjustment of the data to the population distribution. Observed values and their 5%, 50% and 95% percentiles were presented against the 95% confidence/prediction interval corresponding to the same model-predicted percentiles. The pcVPC graphics were obtained with R scripting from Ron Keizer^45^, using the replicas file computed using PhysPK software. Stratification with respect to the normalised dose was applied in the case of the pcVPC plot. Finally, the conditional weighted residual versus time after dose plot was presented as a diagnostic instrument to detect potential model misspecification^46^.

We performed 600 Monte Carlo simulations with estimated population parameters for each TAC model and 3 TAC dose levels (0.025, 0.042, and 0.05 mg/kg, which refer to 1.5, 2.5 and 3.0 mg normalised for 60 kg of body weight) to calculate the mean ${{\rm{AUC}}}_{24}$ associated with each dose. Simulations were performed 36 h and the final 24 h were used, with the aim of ensuring a customised near steady-state for the patients. Covariates and input variables, such as BW, body mass index, sex, age and height, varied according to our paediatric population’s measurements. The bioavailability $F$ (mean ± SD) and the time that the TAC concentration exceeded a gross value of 20 ng/ml as maximum tolerated concentration^47^ were also added to the calculated exposures.

#### Adaptive procedures evaluation

We adapted the models during the second phase (Advagraf data) to correct the prediction errors after the initial fitting with Prograf (day 1). Deviations in the model TAC concentrations could be induced by conversion to Advagraf (day 2) and intrapatient variability during the week. New TAC measurements were taken on day 8 (second monitoring day). The model was adapted using the following procedures (additional details can be seen in the Adapting Evaluation section of the First Supplementary Document):

(i) Procedure 1: The model was first adapted (stage 1) to correct the prediction error of the blood TAC concentrations (C0) measured just before the morning administration of Advagraf on day 8. We employed the WLS adaptive technique using one model parameter ($LibT$ for PBPK and $MTT$ for PK). The parameter adjustment occurred at the instant of TAC formulation conversion (beginning of day 2). We performed a second parametric adaptation (stage 2) to maximise the prediction accuracy of the other TAC measurements during day 8. The parametric change occurred at the beginning of day 8. We applied adaptive Bayesian and WLS techniques in this second stage.
(ii) Procedure 2. We adapted the models to correct the prediction errors in the blood TAC concentrations, using all day 8 measurements simultaneously. The parametric adjustment occurred at the beginning of day 2. We applied adaptive Bayesian and WLS techniques.

The first adaptation of procedure 1 sought to adjust the model parameter that had a greater effect on the drug release rate. After the adjustment had been successfully applied and the first TAC measurement on day 8 had been accurately predicted, the need for a second model adaptation was evaluated to analyse whether there was significant intraindividual parameter variation. The Bayesian technique cannot be applied in procedure 1 - stage 1 because $LibT$ and $MTT$ are not in the set of the models’ population parameters, as defined in the Model analysis subsection (additional details appear in the First Supplementary Document, Adaptive modelling techniques).

We employed a log-trapezoidal integration method to calculate the reference ${{\rm{AUC}}}_{24}$ (NCA) on day 8, which was then compared with the ${{\rm{AUC}}}_{24}$ from the models. The accuracy of the TAC model predictions was quantified by means of the RMSE_*c*_^43^. We compared the results of these 2 procedures, along with the procedures’ effect on the models’ suitability as predictive engines for dosage recommendations.

## Results

The following two subsections present the results for the first phase of the study (Prograf data). The last subsection (Adaptive Models) shows the results for the second phase (Advagraf data).

### Fitting and evaluation of PK model (Prograf data)

Our initial examination of the model confirmed its structural stability, robustness and ability to provide initial estimates of the parameters to be fitted. More details of the analysis appear in the Results section of the First Supplementary Document.

We applied the FOCE technique without covariates to the additive and proportional residual error PK models. The additive residual model obtained fewer RSEs for all mean parameters (${\theta }_{i}$). The two models had similar Ω variance estimates, except for ${\omega }_{Ka}^{2}$, whose additive value was one-third of the proportional value. The two models also had a similar accuracy for the TAC estimations. We therefore selected the additive residual error PK model.

The MVOF obtained with the PK reduced model (without covariates) was −885.13. We applied the covariate forward stepwise approach. The inclusion of the BSA − $C{l}_{d}$ covariate regression exponential model reduced the MVOF to −888.08, whereas the MVOF was virtually unchanged when the $Chb$ − ${V}_{p}$ covariate regression model was added. In addition, the BSA covariate reduced interindividual variance (IIV) by an average of 70% (CV). Given that the reduction in MVOF (2.95) was near 3.84 (statistic significance level of 5%), we decided to accept BSA as one of the model’s covariate. The value of the estimated exponent ${\theta }_{c}$ for exponential correlation (Eq. (9)) was 1.17.

The mean estimated parameter values ${\theta }_{i}$ and associated interindividual variances, together with their RSEs, for the PK TAC model are presented in Table 3.

**Table 3:** Population PK model parameters’ mean and IIV estimates: value (RSE), for Prograf fitting.

Parameter	Units	Value (RSE %)^†^
*Disposition parameters*		
${\theta }_{Cl}$	(L/h)	5.41 (5.7)
${\theta }_{CLd}$	(L/h)	46.5 (7.5)
$${\theta }_{Vc}$$	(L)	23.0 (7.9)
$${\theta }_{Vp}$$	(L)	39.1 (7.2)
*Circadian parameters*		
${\theta }_{{r}_{chr}}$	(pu)	0.29 (6.4)
$${\theta }_{{t}_{chr}}$$	(h)	0.7 (−)
*Absorption parameters*		
${\theta }_{Ka}$	(ml/h)	13.8 (7.3)
$${\theta }_{MTT}$$	(h)	0.72 (5.5)
*Interindividual variabilities*		
${\rm{I}}{\rm{I}}{{\rm{V}}}_{Cl}$	—	0.10 (20)
${\rm{I}}{\rm{I}}{{\rm{V}}}_{Ka}$	—	0.33 (18)
${\rm{I}}{\rm{I}}{{\rm{V}}}_{MT}$	—	0.05 (40)
${\rm{I}}{\rm{I}}{{\rm{V}}}_{chrred}$	—	0.003 (30)
${\rm{I}}{\rm{I}}{{\rm{V}}}_{chrphi}$	—	0.01 (−)
*Residual error variance*		
${\omega }_{r}^{2}$	—	0.027 (3.7)

^†^RSE is calculated through the standard error SE as SE(*θ*)/*θ* · 100 for *θ* and SE$({\omega }_{\theta }^{2})/(2{\omega }_{\theta }^{2})\cdot 100$ for variances.

CV was approximated as $\sqrt{{\omega }_{i}^{2}}$ for small $\eta$ variance (${\omega }_{i}^{2}$), resulting in CV values of 31% for $Cl$, 57% for ${K}_{a}$ and 22% for $MTT$. A first fitting diagnostic was based on shrinkage metric indices^42^. The shrinkage value related to residual error was $\varepsilon$-shrinkage = 0.014 ± 0.349, and the $\eta$-shrinkage parameters were 0.18, −0.08 and 0.17 for $Cl$, ${K}_{a}$, $MTT$, respectively, and 0.66 for ${r}_{chr}$. Values near zero confirm the goodness of fit.

Other relevant values obtained during the Bayesian estimation were $F=18.8\pm 9.7 \%$, ${{\rm{AUC}}}_{24}=200.3\pm 40.7$ ng/ml · h (model-based), ${{\rm{AUC}}}_{24}=201.48\pm 39.27$ ng/ml · h (NCA log-trapezoidal), and Δ${\rm{AUC}}=-\,1.22\pm 4.67$ ng/ml · h. The accuracy of TAC concentration (log-transformed) referred to the whole set of samples was ${{\rm{RMSE}}}_{c}=0.074\pm 0.032$. These metric values support the goodness of fit of the PK TAC model.

Plots of model TAC concentration against TAC measurements for several randomly selected patients and a table that shows a comparison against parameters obtained by the model created by Andreu *et al*.^16^ are presented in the First Supplementary Document. Parameters have similar and reasonable values, with the exception of the peripheral volume ${V}_{p}$, which is 39.1 l in our model against 526.03 l in the Andreu *et al*. model. This issue is addressed in the Discussion section. The two models had similar interindividual dispersion, despite the fact that our PK model was fitted for paediatric patients and includes diurnal and nocturnal TAC distribution. Therefore, our PK model demonstrates greater predictive capability.

**Figure 3:**
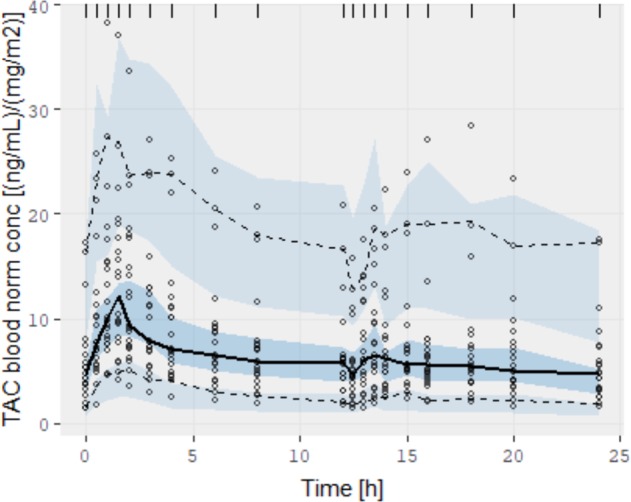
Prediction-corrected visual predictive check of TAC normalised concentration for the PK model. The solid line is the mean observation percentile, and the dashed lines are 5% and 95% observation percentiles. Semi-transparent fields around each observation line represent a simulation-based 95% CI for mean, 5% and 95% model predicted percentiles.

Figure 3 shows the pcVPC based on 500 replicas according to the obtained parametric population. TAC concentrations have been normalised with *D*/BSA mg/m^2^, according to the wide range of TAC dosages for this paediatric population. As shown, the observation percentiles agree with the predicted CIs obtained from PK model simulations. The results for the IIVs agree with those of other studies^16^, despite the studies not requiring TAC predictions during 2 sequential 12-h Prograf administrations.

Additional studies, showing the fulfilment of a normal distribution assumption and the results of a Monte Carlo simulation with 600 runs for the PK model with 3 TAC normalised doses can be found in the First Supplementary Document (PK results). According to the Monte Carlo study, a dose of 0.042 mg/kg achieves an ${{\rm{AUC}}}_{24}$ exposure of 271.4 ± 196.7 ng/ml · h. The high dispersion of ${{\rm{AUC}}}_{24}$ emphasises the need to use MIPD to customise the TAC dose.

### Fitting and evaluation of PBPK model (Prograf data)

Our initial exploration of the PBPK model confirmed its structural stability and robustness, which also provided the initial estimates for the parameters to be fitted. More details of the analysis can be found in the Results section of the First Supplementary Document.

The accuracy of the TAC concentrations was similar with the residual and proportional error, as well as IIV estimates; we therefore selected the additive residual error. Table 4 presents the mean estimated parameter values ${\theta }_{i}$ and their interindividual variances (along with their RSEs) calculated for the PBPK model using the FOCE technique.

**Table 4:** Population PBPK model parameters’ mean and IIV estimates: value (RSE), for Prograf fitting.

Parameter	Units	Value (RSE %)^†^
*Absorption and RBC binding*		
${\theta }_{CLih}$	(1/h)	956.6 (6.1)
${\theta }_{Bmax}$	(*μ*g/L)	11.0 (6.3)
${\theta }_{Kd}$	(*μ*g/L)	1.6 (9.0)
*Circadian parameters*		
${\theta }_{{r}_{chr}}$	(pu)	0.16 (8.3)
$${\theta }_{{t}_{chr}}$$	(h)	0.7 (−)
*Interindividual variabilities*^§^		
${\rm{I}}{\rm{I}}{{\rm{V}}}_{CLih}$	—	0.114 (16)
${\rm{I}}{\rm{I}}{{\rm{V}}}_{Bmax}$	—	0.004 (16)
${\rm{I}}{\rm{I}}{{\rm{V}}}_{Kd}$	—	0.083 (17)
${\rm{I}}{\rm{I}}{{\rm{V}}}_{{r}_{chr}}$	—	0.367 (32)
${\rm{I}}{\rm{I}}{{\rm{V}}}_{{t}_{chr}}$	—	0.01 (−)
*Residual error variance*		
${\omega }_{r}^{2}$	—	0.037 (7.4)

^†^RSE is calculated through the standard error SE as SE(*θ*)/(*θ*) · 100 for *θ* and SE$({\omega }_{\theta }^{2})/(2{\omega }_{\theta }^{2})\cdot 100$ for variances.

The CVs were calculated in the same manner as in PK model, with values 33% for $C{l}_{ih}$, 6.3% for ${B}_{max}$ and 28.8% for ${K}_{d}$. Shrinkage index for residual error was $\varepsilon$-shrinkage = 0.039 ± 0.30, and $\eta$-shrinkages were −0.32, 0.80 and 0.72 for $C{l}_{ih},{B}_{max},{K}_{d}$, respectively, and 0.12 for ${r}_{chr}$. Values near zero confirm the goodness of fit^42^.

Additional values obtained during the Bayesian estimation were $F=15.0\pm 1.6 \%$, ${{\rm{AUC}}}_{24}=198.3\pm 41.3$ (model based) and ${{\rm{AUC}}}_{24}=201.48\pm 39.27$ (NCA log-trapezoidal), with Δ${\rm{AUC}}=-\,3.13\pm 6.37$ ng/ml · h. The accuracy of the TAC concentrations (log-transformed) with respect to TAC measurements was ${{\rm{RMSE}}}_{c}=0.092\pm 0.041$. These metric values support the goodness of fit of the PBPK model. Plots of PBPK model TAC concentration versus TAC measurements for randomly selected patients and the comparison with parameters from other sources can be found in the First Supplementary Document.

**Figure 4:**
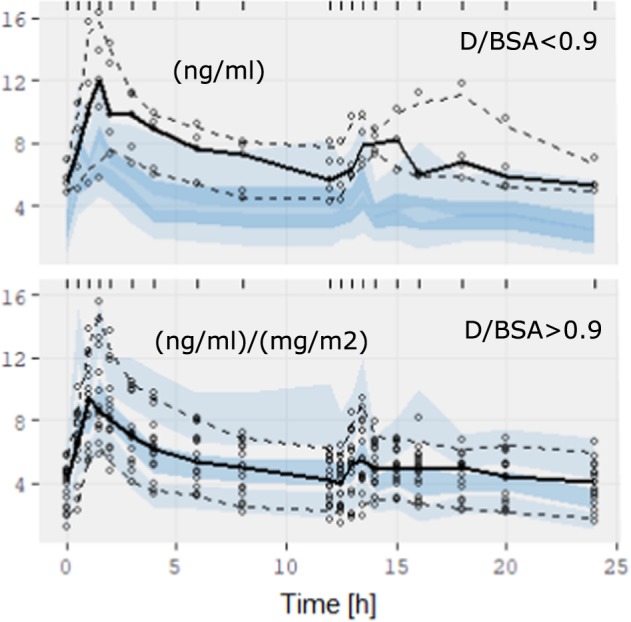
Dose stratified (D/BSA mg/m^2^) prediction-corrected visual predictive check of TAC concentration for the PBPK model. The solid line is the mean observation percentile, whereas dashed lines are the 5% and 95% observation percentiles. Semi-transparent fields around the observation lines refer to simulation-based 95% CIs for mean, 5% and 95% model predicted percentiles.

Figure 4 presents the pcVPC based on 500 replicas for the fitted PBPK model. A low TAC dose (*D*/BSA < 0.9 mg/m^2^) is presented in the upper plot with nonscaled concentrations (ng/ml). A non-low TAC dose (*D*/BSA ≥ 0.9 mg/m^2^) is shown in the bottom plot, using scaled concentrations (C/(D/BSA).

The non-low TAC dose pcVPC shows very good agreement between the predicted CIs obtained from PBPK model simulations and observations. More than 75% of patients pertain to this case. The low-dose plot is based on nonscaled concentrations to avoid amplifying the graphical distances due to low denominator values (D/BSA mg/m^2^). The model-observations agreement is not as good in this case. However, the 95% CI of the model-predicted percentiles was much smaller in the PBPK model than in the PK model (see Fig. 3), which demonstrates the PBPK model’s high predictive capability.

Standard metric plots that confirm the normal distribution assumption and results of a Monte Carlo study with 600 runs and 3 TAC doses per BW can be found in the PBPK results of the First Supplementary Document. The ${{\rm{AUC}}}_{24}$ exposure for a dose of 0.042 mg/kg is 177.6 ± 50.9 ng/ml · h. The deviation is smaller than in the PK model but still significant, which supports the need for MIPD in customising the TAC dose.

### Adaptive models (Advagraf data)

**Figure 5:**
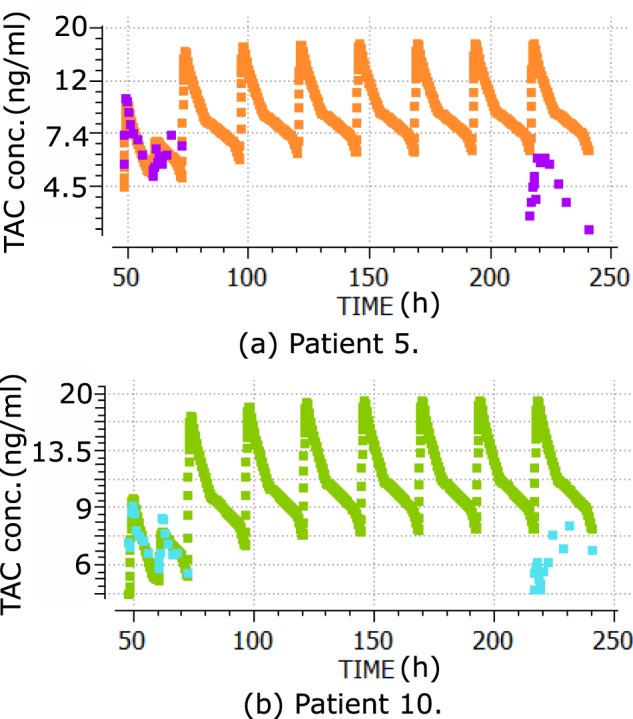
Central compartment TAC concentrations from the PK model fitted for Prograf data (days 1–8), versus blood TAC measurements used for model fitting (day 1) and blood TAC measurements during hospital re-admission (day 8) for Patient 5 (**a**) and Patient 10 (**b**). Time in h.

Figure 5 shows the blood TAC concentrations predicted by the PK model fitted with Prograf data and with no parametric adjustment from day 1 to day 8, for 2 randomly selected patients. The TAC formulation changed from Prograf to Advagraf at the beginning of day 2. Two daily TAC doses (Prograf) on day 1 and once-daily doses (Advagraf) on days 2–8 were considered during the model’s execution. The model TAC concentration at the beginning of day 8 (re-admission) (C0) overvalued the C0 TAC measurement in 19 of 21 patients. The differences measured minus the model C0 for the whole population was −1.91 ± 1.46 ng/ml (mean ± SD).

**Figure 6:**
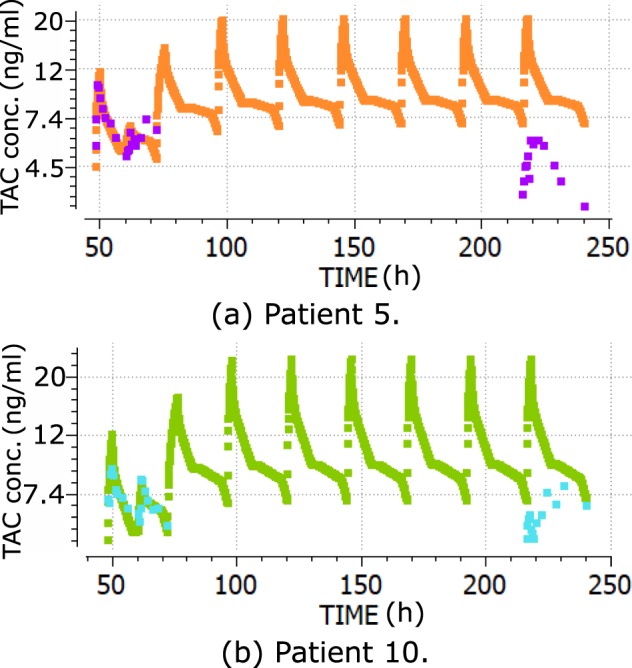
Blood TAC concentrations from the PBPK model fitted for Prograf data (days 1–8), versus blood TAC measurements used for model fitting (day 1) and blood TAC measurements during hospital re-admission (day 8) for Patient 5 (**a**) and Patient 10 (**b**). Time in h.

Figure 6 shows the same prediction scenario with the fitted PBPK model. Prediction errors were similar. The differences measured minus the model C0 for the whole population was −2.94 ± 2.93 (mean ± SD ng/ml).

The TAC overvaluation could be due to the fact that the models were not adjusted to consider the prolonged release of Advagraf. However, errors in TAC predictions can also be induced by intraindividual variations in the parameters over the course of a week. Adaptive procedure 1 was defined to discriminate those potential causes. Models were first adapted to correct the influence of TAC formulation, using a parameter associated with the drug release rate. Once the model prediction of TAC concentration at the beginning of day 8 is accurate, any additional adaptation required to correct other TAC concentration predictions during day 8 would support the existence of intraindividual parameter variations.

Procedure 1 requires the existence of a model parameter related to the drug release rate. Procedure 2, however, uses an alternative adaptive technique that avoids this requirement. Adapted models provided by procedure 2 are compared with final adapted models produced by procedure 1.

#### Adaptive procedure 1

Procedure 1 was unable to adapt the PK model by means of the $FDT$ parameter for providing an accurate C0 TAC concentration. However, this adaptive procedure was successfully applied to the PBPK model to correct C0 TAC concentration. This result demonstrates that the drug liberation time $LibT$ (and the underlying mechanism) is correlated with drug formulation release, in contrast to $FDT$ in the PK model.

The predictive accuracy of the PBPK model with adjusted $LibT$ (WLS adaptive technique) was ΔC0 (C0 measurement - model difference), equal to 0.004 ± 0.039 (mean ± SD ng/ml) for the whole population. $LibT$ increased 176 ± 225.4 min (20 of 21 patients showed increased $LibT$ value). The mean $LibT$ increment agrees with the greater release rate of Advagraf. However, the high dispersion suggests there could be other parameters with significant intrapatient variation. Plots with predictions of the adapted PBPK model for randomly selected patients and additional details are presented in adaptive procedure 1 in the First Supplementary Document.

Despite the accurate prediction of trough TAC on the re-admission day, the $LibT$-adapted PBPK model did not provide an accurate prediction of the other TAC measurements on that day. Table 5 shows the adjustment of parameters in the second stage of adaptive procedure 1. The C0-instant column provides differences in parameters between consecutive patients before this second model adaptation.

**Table 5:** Parameter increments due to the second adjustment of the PBPK model (adaptive procedure 1), with Bayesian and WLS techniques.

	C0-instant^†^	Bayes	WLS
Δ*Cl*_*ih*_ (1/h)	−4.88 ± 563.8	143.2 ± 380.6	−169.0 ± 333.6
Δ*B*_*max*_ (ng/ml)	0.0 ± 0.0	0.3 ± 1.2	—
Δ*K*_*d*_ (ng/ml)	0.0 ± 0.1	−0.2 ± 0.4	—
Δ*K*_*abs*_ (1/h)	—	—	0.4 ± 2.7
Δ*LibT* (min)	2.9 ± 326.8	—	12.3 ± 343.7
ΔAUC_24_^‡^ (ng/ml · h)	—	0.0 ± 16.3	0.8 ± 6.7
RMSE_*c*_	—	0.157 ± 0.070	0.088 ± 0.032

ΔAUC_24_ (difference model − NCA AUC_24_), RMSE_*c*_ (log-transformed blood concentration error model - measurement, mean ± SD).

^†^Differences in C0-instant calculated as inter-individual differences of parameters. ^‡^NCA value calculated with log-trapezoidal integration.

TAC concentrations and ${{\rm{AUC}}}_{24}$ predictions of the second adapted PBPK model were accurate, as shown. The main parameter involved in the final model adaptation is the intrinsic hepatic clearance per liver volume ($C{l}_{ih}$). Other parameters, such as ${K}_{abs}$ in WLS and ${B}_{max},{K}_{d}$ in Bayes, experienced lower variations, other than having a smaller influence on TAC concentrations, as proven in the sensitivity analysis. $LibT$ dispersion is related to the re-adjustment of the parameters with relevant intraindividual variation. The mean interindividual differences are approximately zero, and intraindividual dispersions are smaller than interindividual dispersions.

The main conclusions from the evaluation of adaptive procedure 1 are the confirmation that the $LibT$-associated mechanism governs the release rate of the TAC formulation and thus may be adjusted through adaptive techniques such as WLS. We also found non-negligible IPVs for $C{l}_{ih}$ and ${K}_{abs}$. Additional plots and data in the First Supplementary Document show more details that support these conclusions.

#### Adaptive procedure 2

Table 6 shows the parametric adjustment of the PBPK model using adaptive procedure 2. The accuracy of TAC predictions, given by RMSE_*c*_ and ΔAUC_24_, were better for WLS than Bayesian adaptive technique.

**Table 6:** Parameter increments for the PBPK model adjusted according to adaptive procedure 2.

	Prograf^†^	Bayes	WLS
Δ*Cl*_*ih*_ (1/h)	−4.88 ± 563.8	637.1 ± 421.6	−38.3 ± 325.8
Δ*B*_*max*_ (ng/ml)	0.0 ± 0.0	0.2 ± 0.3	—
Δ*K*_*d*_ (ng/ml)	0.0 ± 0.1	−0.2 ± 0.4	—
Δ*K*_*abs*_ (1/h)	—	—	2.3 ± 3.5
Δ*LibT* (min)	—	—	277.2 ± 284.4
Δ*F* (%)	_0.1 ± 12.1_	10.0 ± 1.6	−1.8 ± 6.4
ΔAUC_24_ (ng/ml · h)	—	4.5 ± 15.6	2.8 ± 8.6
RMSE_*c*_	—	0.235 ± 0.095	0.109 ± 0.063

Final rows show the increment of bioavailability of day 8 with respect to day 1, *F*, as well as the accuracy indices ΔAUC_24_ (difference model − NCA value) and RMSE_*c*_ (log-transformed, mean ± SD).

^†^Differences Δ in Prograf column are calculated as inter-individual differences of parameters.

The results agree with those of procedure 1. $LibT$, $C{l}_{ih}$ and ${K}_{abs}$ remain the primary parameters that need to be adapted due to the change in formulation release and IPV. ${K}_{abs}$ shows a higher mean increase (2.3 1/h) than in procedure 1. However, adaptive procedure 1 included 2 sequential changes in $LibT$ (the first value acts on days 2–7, while the second value acts on day 8), against the unique $LibT$ change in procedure 2 (acting during days 2–8). This explains the IPV differences between procedures 1 and 2.

The main conclusions form the evaluation of procedure 2 when compared with procedure 1 for the PBPK model are the confirmation of the IPV of $C{l}_{ih}$ and ${K}_{abs}$ parameters and the demonstration that $LibT$ can support changes in the release rate of the TAC formulation. $LibT$ cannot be adjusted through the Bayesian adaptive method because it is not in the set of PBPK model population parameters, which explains its poorer predictive behaviour. Additional plots in the First Supplementary Document show more details about this poorer behaviour. Differences in model-based bioavailability between day 1 and day 8 are addressed in the Discussion section.

Table 7 shows the parametric adjustment of the PK model using adaptive procedure 2. The $Cl$ and ${K}_{a}$ means were reduced with respect to their previous values in the Prograf fitted model. The $FDT$ mean was slightly increased in agreement with the prolonged release of Advagraf, but dispersion was high. The mean transit time ($MTT$) was virtually unchanged. The accuracy of the predictions (provided by RMSE_*c*_ and ΔAUC_24_) were better for WLS than for the Bayesian adaptive method. This behaviour can be explained because $FDT$ is not a PK model population parameter. The difference in accuracy between the Bayesian and WLS methods is smaller than for the PBPK model, consistent with the lower influence of FDT than LibT on the model’s behaviour.

**Table 7:** Parameter increments for the PK model adjusted according to adaptive procedure 2.

	Prograf^†^	Bayes	WLS
Δ*Cl* (L/h)	−0.02 ± 2.08	−1.33 ± 1.55	−1.93 ± 1.80
Δ*K*_*a*_ (ml/h)	1.23 ± 13.93	−9.91 ± 8.10	−9.62 ± 8.41
Δ*MTT* (h)	0.00 ± 0.21	0.055 ± 0.126	−0.038 ± 0.215
Δ*FDT* (h)	—	—	0.34 ± 0.50
Δ*F* (%)	1.1 ± 15.0	2.2 ± 10.0	−3.6 ± 12.6
ΔAUC_24_ (ng/ml · h)	—	−6.51 ± 8.74	−3.76 ± 8.11
RMSE_*c*_	—	0.096 ± 0.037	0.085 ± 0.036

Final rows show the increment of bioavailability between days 8 and 1, *F*, in addition to the accuracy indices ΔAUC_24_ (difference model − NCA value) and RMSE_*c*_ (log-transformed, mean ± SD).

^†^Differences Δ in Prograf column are calculated as inter-individual differences of parameters.

The intraindividual dispersion (SD) of the adjusted parameters for adaptive procedure 2 were lower than the interindividual dispersion obtained during Prograf fitting for the PK and PBPK models, which supports the robustness of the models and adaptive techniques.

## Discussion

This study has developed and investigated 2 new TAC population PK models with the aim of evaluating their capability as predictive engines for personalised dosage recommendations. One of the models followed a PBPK approach, while both models added circadian modulation of absorption and clearance variables, which, to the best of our knowledge, has not been presented in any published PK TAC model to date, despite TAC chronomodulation having been widely cited^7,22,23^.

The study was divided into 2 phases. The first phase fitted the models against a paediatric population with twice daily Prograf administration (Prograf data). The second phase evaluated their ability to predict effects of changes in TAC formulation (Advagraf data) and intrapatient variation, through two adaptive techniques.

Based on the results from the first phase, the PK and PBPK models demonstrated robustness against parameter perturbations, as well as accuracy and suitability for describing blood TAC dynamics for paediatric renal transplant recipients administered Prograf every 12 h. The inclusion of circadian rhythms improved the models’ predictive capability, measured with 24-h blood TAC concentrations and administering Prograf twice daily.

Both the IIV and residual variability (see Table 3 in the First Supplementary Document) of the new PK model were similar to those of the Andreu *et al*. model^16^, despite the fact that the new PK model did not consider IOV. This outcome suggests that the circadian rhythms explain a significant portion of IPV associated with IOV in the Andreu *et al*. model. In addition, IIV and residual variabilities were based on a 24-h TAC measurement period in our study, in contrast to the 12 h of the Andreu *et al*. study. Considering the greater heterogeneity of our paediatric renal transplant recipients, these results suggest that the new PK model has greater predictive capability than the Andreu *et al*. PK model.

The parametric comparison between our PK model and the Andreu *et al*. PK model was addressed in the previous section, although the mean peripheral volume (${V}_{p}$), requires additional discussion. The ${V}_{p}$ was 39.1 l in our model compared with 526.03 l in the Andreu *et al*. model. The authors justify their value based on the extensive TAC distribution in tissues, an assertion that appears to conflict with the published steady state distribution volumes of TAC, which are approximately 1300 l for plasma and 47.6 l for whole blood (for healthy individuals)^48^. The TAC concentrations in our model and in the Andreu *et al*. model refer to whole blood, in which a distribution volume of approximately 40–100 l appears more reasonable. A possible solution is that the ${V}_{p}$ in the Andreu *et al*. study refers to the apparent value (${V}_{p}/F$).

Other 2-compartment PK models of TAC give the following values of ${V}_{p}$ (calculated with $F\approx 10 \%$ in the case of apparent values): 152 l (paediatric)^15^, 63.6 l (adults)^13^, 47.7 l (adults)^17^. In the Benkali *et al*. study^17^ ${V}_{p}$ was calculated as ${K}_{cp}/{K}_{pc}\cdot {V}_{c}$, in which flow micro-constants ${K}_{cp},{K}_{pc}$ and central volume ${V}_{c}$ were provided by the authors.

The PBPK model showed 95% CIs for the predicted percentiles for TAC concentrations much smaller than in the PK model, as well as ${{\rm{AUC}}}_{24}$ dispersions for normalised TAC doses 4-fold lower than in the PK model (Monte Carlo studies, see Tables 4 and 6 in the First Supplementary Document). These results demonstrate the better predictive behaviour of the PBPK model compared with the PK model for the first phase of the study, consistent with the mechanistic nature of PBPK models^18^.

The poorer agreement between the model and the observations for low doses (D/BSA < 0.9 mg/m^2^, Fig. 4) in PBPK needs clarifying. This behaviour was not observed in the PK model because it had much higher interpatient concentration variabilities than the PBPK model. No demographic differences were found in patients with low doses. Cho *et al*. determined the accuracy of the TAC measurements by performing a dimension TAC assay, which showed a total CV accuracy in the range of 7.3–5.7% for TAC concentrations of 4.09–18.5 ng/ml; a limit of blank of 0.29 ng/ml, a limit of detection of 0.47 ng/ml and a limit of quantification of 0.81 ng/ml, as well as a carryover between samples of 0.41% and an average correlation coefficient with respect to the liquid chromatography-tandem mass spectrometry method of 0.8165^49^. However, the correlation coefficient was 0.6347, with a bias of 0.277 (−1.205 to 1.759) ng/ml for kidney transplant recipient measures, which could have contributed to the poorer behaviour for low doses because of the lower TAC concentrations (associated with an increase in the total analytical assay CV). Trough blood TAC concentrations were 6.5 ± 1.3 ng/ml for the total population versus 5.74 ± 0.9 ng/ml for the low-concentration group, which agrees with that suggestion that lower TAC concentrations contributed to the poorer behavior for low doses. The small number of patients in the low-dose group (5) limits additional research in this study. However, whole blood TAC concentration PBPK predictions for low-dose patients (${{\rm{RMSE}}}_{c}=0.085\pm 0.033$) maintain the same accuracy as for the total population (${{\rm{RMSE}}}_{c}=0.092\pm 0.041$, see fitting PBPK model in the Results section), supporting the suitability of the PBPK model for the low-dose group.

This is the first pharmacokinetic model-based study that supports the relationship between daily TAC concentration patterns and the circadian modulation of clearance and absorption, which has been previously suggested^23^. This knowledge is relevant, given the fact that circadian variations in TAC pharmacokinetics have important clinical implications^7,22,23^. To the best of our knowledge, this is also the first study to compare a reduced PBPK model with a PK model for the same clinical population (paediatric) subjected to TAC administration.

In the second phase of the study, both models required adjustment using adaptive techniques to accurately predict blood TAC concentrations during the hospital re-admission of patients on day 8, after the switch from the Prograf to Advagraf formulation on day 2.

The increase in $LibT$ in the PBPK model, using the WLS adaptive technique, corrected the deviation of the predicted trough TAC concentration (C0) at the beginning of day 8. The mean value of the adapted $LibT$ was $100+176=276$ min, in which 100 min is the value estimated in the Prograf phase and 176 ± 225.4 min is the parameter adjustment (stage 1 of procedure 1). An estimated value of $LibT$ can also be obtained according to the liberation mechanism implemented in the model, considering that 40% of the Advagraf mass is released approximately 1.5 h later in simulated gastric fluid^50^, using Eq. 7 in the First Supplementary Document, which states:

10 $$\frac{1}{LibT}=\frac{{K}_{lib}}{D}=\frac{0.4}{1.5\cdot 60}=4.4\cdot {10}^{-3}\,{{\rm{\min }}}^{-1}$$

Therefore, $LibT=1/4.4\cdot {10}^{-3}=225$ min, which is near the mean of 276 (176 + 100) min obtained by the adaptive technique with TAC measurements from day 8. This result supports the strong relationship between the drug liberation time ($LibT$) and the drug formulation release and demonstrates the capability of the WLS adaptive technique and PBPK model to predict blood TAC concentrations after changes in the drug formulation release rate.

The requirement of a new parametric adjustment in the PBPK model to accurately predict blood TAC concentrations during day 8 demonstrates the existence of a significant IPV in $C{l}_{ih}$ and ${K}_{abs}$ over a week of progression, as well as circadian rhythms. This outcome is important for 2 reasons. First, our clinical study was designed to achieve high pharmacological adherence and control food intake, DDI, diarrhoea and the TAC formulation. The selected analytical assay, based on the affinity chrome-mediated immunoassay, fulfils the recommendations of the International Association of Therapeutic Drug Monitoring and Clinical Toxicology (total accuracy CV < 9%), with good scores for the detection limits, linearity and carryover^49^. This finding confirms that the control of these conditions is not enough to avoid TAC IPV in stable kidney transplant recipients^51^. Second, this is the first study to calculate TAC IPV by means of parametric intrapatient changes, instead of the standard scaled TAC concentrations (C0/D)^7,8^, which provides a method for analysing the mechanisms are involved.

Although the PK model cannot be adapted using the parameter $FDT$ to correct C0 TAC due to the low correlation of $FDT$ with the drug release rate, both the PK and PBPK models could be adjusted to correct the predictions of TAC concentrations on day 8, using the strategy of adaptive procedure 2. Adjustments to the PBPK model with procedure 2 agree with those of procedure 1, supporting the reliability of procedure 2 as an adaptive technique to adjust PK models. These results support the suitability of population PK and PBPK models as predictive engines for personalised dosage recommendations.

Table 8 presents measured and calculated exposures ${{\rm{AUC}}}_{24}$ for Prograf (day 1) and Advagraf (day 8, 1 week after 1:1 conversion). Associated $F$ bioavailability can be calculated from $F\cdot D=Cl\cdot {\rm{AUC}}$ for constant $Cl$^52^. Although this is not the case due to IPV in $Cl$, Eq. (2) in our model solved this issue. Tables 6 and 7 show the changes in $F$ values after TAC formulation conversion. Despite a mean reduction in ${{\rm{AUC}}}_{24}$ of approximately 20 ng/ml · h after 1:1 conversion, in agreement with previous studies^37,53^, $F$ bioavailability calculated with WLS-adapted PK and PBPK models experienced a small mean variation with large dispersion. This result demonstrates that $F$ bioavailability is not correlated with ${{\rm{AUC}}}_{24}$ in 1:1 Prograf to Advagraf conversion due to the IPV of TAC clearance. An additional plot that shows $F(t)$ in the PBPK model as a function of $LibT$ can be found in the First Supplementary Document.

**Table 8:** TAC AUC_24_ (ng/ml · h) values (mean ± SD) at days 1 (Prograf) and 8 (Advagraf) calculated through measurements and models.

	NCA^†^	PK^‡^	PBPK^‡^
AUC_24_ day 1	201.48 ± 39.27	200.3 ± 40.7	198.3 ± 41.3
AUC_24_ day 8	177.1 ± 42.3	177.4 ± 48.0	179.9 ± 42.3

^†^Log-trapezoidal integration with blood measurements. ^‡^WLS - adapted models (procedure 2).

To summarise, after highlighting the need to further the development of real-time MIPD for TAC that lowers the risk of graft failure in kidney transplantation, this study presented a comparison in the predictive performance of 2 new TAC PK models, with 3 major conclusions. First, TAC absorption and metabolic clearance circadian rhythms appear to be the major cause of daily TAC IPV and can be modelled with a population approach to improve the accuracy of prediction, mainly with PBPK. Second, TAC PK models may be used as adaptive predictive engines for MIPD, under various TAC formulations. The PBPK approach has demonstrated better adaptive capability than single PK. Third, parametric changes due to TAC IPV can be calculated during the adaptive process, which provides information on the mechanisms involved. A preliminary real-time MIPD software design for clinical practice based on PK models has been published^54^ and provides guidelines for subsequent research.

The conclusions of this study are limited to the controlled and stable renal transplant paediatric population. Mathematical models are limited to the functional scope defined in the Model Development section. The interoccasion variability was not implemented in our models as justified in the Methods Section, despite this term is commonly included in population PK models and it can affect the TDM accuracy. However, our outcomes suggest that circadian rhythms explain a significant portion of IPV associated with IOV. In addition, the exclusion of IOV term may be a preferred approach to maximize precision in the calculation of individual doses using model-based Bayesian forecasting, according to the recent study by Abrantes *et al*.^55^. Moreover, adaptive mathematical models have demonstrated their ability to predict customized biochemical levels, such as glycemia, without the inclusion of IOV^25^. We did not consider DDIs at the present time. Further research is needed to add unstable patients and extend models to consider DDIs. Current knowledge on physiological and pharmacokinetic modelling suggests that these extensions are feasible.

## Supplementary information

Supplementary Information 1

Supplementary Information 2

## Data Availability

All data generated or analysed during this study are included in this published article (and its Supplementary Information files).
