# Supplementary material for: Predictive engines based on pharmacokinetics modelling for tacrolimus personalized dosage in paediatric renal transplant patients
Source: Sci Rep. 2020 May 5;10:7542. doi: 10.1038/s41598-020-64189-9 (PMC7200804; doi:10.1038/s41598-020-64189-9)
Supplement: Supplementary file 1 — Supplementary Information 1 [file 41598_2020_64189_MOESM1_ESM.pdf]

Supplementary

Predictive engines based on pharmacokinetics modelling for tacrolimus personalized dosage in paediatric renal transplant patients

Manuel Prado-Velasco Alberto Borobia
Antonio Carcas-Sansuan

Models development and adaptive techniques

Pharmacokinetics model

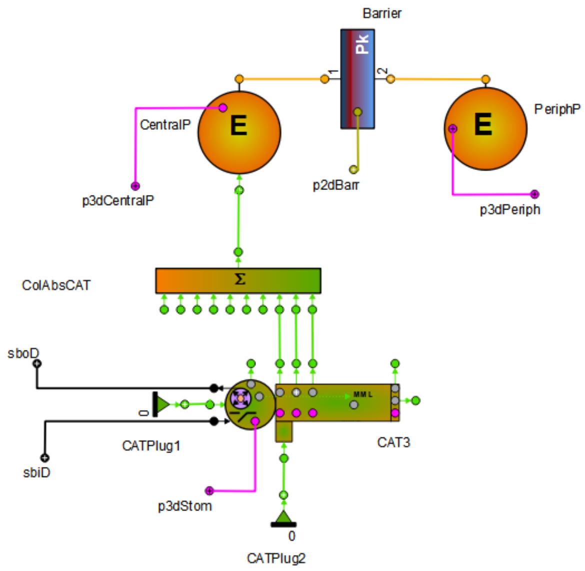

Figure 1: PhysPK diagram of the PK model for TAC with a 2-compartment structural main system (CentralP, Barrier, PeriphP) and a 3-compartment transit and absorption (ColAbsCAT, CAT3) subsystem.

The pharmacokinetic (PK) model is described by a 2-compartment structural system and a 3-compartment transit and absorption subsystem (CAT3). Figure 1 shows a diagram of the model built with PhysPK. The structural system is composed of compartments CentralP (Central) and PeriphP (Peripheral), with a Barrier element controlling the TAC flow rate between them. The CAT3 system is shown at the bottom of the diagram and is connected with the Central compartment through a collector that sums the TAC absorptions from the 3 sections of the small intestine. Lines connecting cited elements in this diagram represent TAC mass flow rates

The remaining lines that connect PK model elements with circular connecters, such as p3dCentralP (CentralP), are provided by the simulation tool to facilitate access to the variables inside. For instance, a computational integrator component can access the elimination TAC mass flow rate from the Central compartment and calculate the accumulated elimination TAC or access the TAC concentration and calculate the area under the curve (AUC). The diagram is provided to clarify the model's scope and is governed by the following

equation system (1):

$$
\begin{aligned}
V_c \frac{\mathrm{d}C_c}{\mathrm{d}t} &= K_{achr}(t) \sum_{j=1}^{j=3} C_{si,j} - Cl_{chr}(t)C_c + Cl_d(C_p - C_c) \\
V_p \frac{\mathrm{d}C_p}{\mathrm{d}t} &= -Cl_d(C_p - C_c) \\
\frac{\mathrm{d}M_s}{\mathrm{d}t} &= D_f - k_t M_s \\
\frac{V_{si}}{3} \frac{\mathrm{d}C_{si,j}}{\mathrm{d}t} &= \phi_{t,j-1} - K_{achr}(t)C_{si,j} \\
M_{si,j} &= \frac{V_{si}}{3} C_{si,j} \\
\frac{\mathrm{d}M_c}{\mathrm{d}t} &= \phi_{t,3} \\
\phi_{t,0} &= k_t M_s \\
\phi_{t,j} &= k_t M_{si,j}
\end{aligned}
\tag{1}
$$

The variables are defined in the main text of the article. Index $j = 1, 2, 3$ refers to the small intestine section number. Additional variables used in equation (1) include TAC masses in the stomach, small intestine section j, and colon: $M_s, M_{si,j}$ and M_c, as well as the TAC transit flow rate from the stomach, $\phi_{t,0}$ and from small intestine section j, $\phi_{t,j}$.

Gastrointestinal fluid volumes (CAT volumes) are defined as $V_{cat,j} = V_{cat} f_j$, in which V_{cat} is the total CAT volume, and f_j is the volume fraction of the j zone, which were calculated as $V_{cat} = 0.475$ l, and $f_s = 0.84$ (stomach), $f_{si} = 0.13$ (small intestine) and $f_c = 1 - f_s - f_{si}$ (colon), according to (Schiller et al., 2005) . Absorption from the small intestine to the central compartment has first-order kinetics, with rate constant $k_{ta} = K_a/V_{si}$, where K_a (ml/h) is the absorption constant and V_{si} (ml) is the small intestine fluid volume.

Linear assumptions in central clearance and gastrointestinal transit were compared with saturable (Michaelis-Menten) assumptions using the parametric-fitting methodology stated in the Methods section, with no improvements in TAC predictions or interindividual parameter variability.

The absolute sensitivity of TAC concentrations with respect to changes in CAT volume fractions f_j and in the small intestine MTT was much less than 1, confirming the robustness of the model with respect to those parameters. This robustness is a desired feature in the PK model, given the high variability of gastrointestinal fluid volumes and mean transit times (Kimura and Higaki, 2002).

Circadian transitional zones in $f_w(t)$ ensure a smooth change between diurnal and nocturnal phases. The durations of the diurnal and nocturnal phases were assumed to be equal, and transitions were estimated to be 20% of the phase durations. A sensitivity analysis of TAC concentrations with respect to changes in duration parameters of the circadian waveform function confirmed the robustness of the model (absolute sensitivity values < 1). Model parametric fitting

was also performed with various transition durations with no improvements in TAC predictions either interindividual variability

The drug administration subsystems of the PK model include the drug form delivery time FDT (h), which is the time required for TAC to be fully available for transit. This was calculated at 0.083 h (5 min) in a fast-release formulation such as Prograf. Absolute values for TAC sensitivity with respect to FDT were much less than 1, confirming the robustness of the model in terms of FDT perturbations

The time-dependent bioavailability $F(t)$ is equal to the F value calculated with the standard equation $F \cdot D = \text{AUC} \cdot Cl$ when Cl is constant. On the other hand, the mean value of $F(t)$ converges to the standard bioavailability F for sufficiently long-term values (several administration periods)(Prado-Velasco, 2018).

Physiologically based pharmacokinetic model

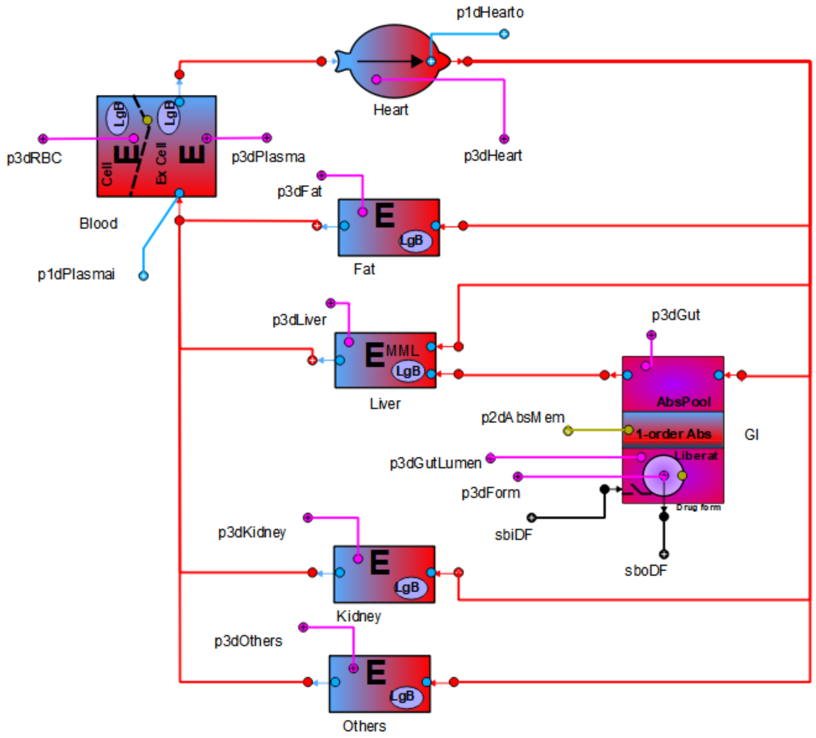

Figure 2: PhysPK diagram of the TAC PBPK model with 4 flow-limited tissues (fat, kidneys, liver, other) and 2 membrane-limited tissues (gastric and blood).

Additional model data. The oil - water partition coefficient (log) for TAC was assumed to be 3.3 (DrugBank, 2018). The partition coefficient of other tissues was considered equal to that of muscle tissue due to the nearly identical relationship observed between muscle and other body tissues (Poulin and Theil, 2000).

Tissue volumes are proportional to body weight (BW) based on ratio values obtained from published physiological databases (ILSI, 1994), except for the fat volume ratio P_f (l/kg of BW), which was calculated though a correlation for children and extracted from Table 3 in (Deurenberg et al., 2007) to give:

$$P_f = (1.51 \cdot \text{BMI} - 0.70 \cdot \text{age} - 3.6 \cdot \text{sex} + 1.4)/100\,, \tag{2}$$

in which age is given in years, sex is 1 (males) or 0 (females), standard body mass index (BMI) is in kg/m2 units and BW is in kg. Fat volume is then $V_f = P_f \cdot \text{BW} \cdot 10^3$ (ml). Paediatric scaling for this reduced physiologically based pharmacokinetic (PBPK) model was completed by considering changes in water and lipid fractions in fat tissue according to Price et al. (2003). Exploratory simulations have shown that paediatric fat scaling is required to achieve optimal TAC concentration predictions, whereas scaling of other model tissues is not needed. This conclusion agrees with the largest changes in total volume, water, and lipid fractions of adipose tissue during ontogeny (Price et al., 2003).

No other ontogeny scaling was implemented, given the objective of this study was to assess a reduced PBPK model as a predictive engine for a real-time model-informed precision dosing. Variables associated with gut absorption, liver metabolism and red blood cell binding (which are related to proteins with a significant influence on the accuracy of TAC distribution predictions) have been defined as population parameters to be fitted.

Table 1: Physiological values for tissues in the PBPK model extracted from (ILSI, 1994; Pelekis et al., 1995; Poulin and Theil, 2000). See main text for nomenclature and units.

Tissue	$P_i^{\S}$	W_f	nL_f	phL_f	q_i
Liver	1350/70	0.75	0.03	0.03	17.1
Kidney	280/70	0.78	0.02	0.02	14.3
Gut	2100/70	0.72	0.05	0.02	12.8
Fat	†	0.39	0.57	$1.45 \cdot 10^{-3}$	7.9
Others	†	0.76	0.0338	0.0062	20.7
Blood	4500/70	–	–	–	‡

‡ Blood perfusion flow rate is the sum of the Liver, Kidney, Fat and Others tissues' blood perfusion flow rates.
§Index i refers to tissue. † P_f and P_o are calculated in text.

Table 1 shows physiological data related to PBPK tissues. P_i (ml/kg) provides the volume of tissue i through the equation $V_i = P_i \cdot \text{BW}$ (ml), in which BW is in kg. Index i takes values l (liver), k (kidney), g (gut), f (fat) or o (others). P_f does not appear since it is found in equation (2). Gut lumen

volume was considered equal to gut tissue volume. *Others* tissue volume was $V_o = \mathrm{BW} \cdot 10^3 - \sum_i V_i$ (ml), in which the sum is extended by liver, kidney, gut tissue, fat and blood. The value P_o is equal to V_o/BW. The mean body mass density was considered 1 g/L as is usual in PBPK (ILSI, 1994). Heart volume $V_h = 150/70 \cdot \mathrm{BW}$ ml refers only to the heart chamber. Plasma volume (V_p) and red blood cell (RBCs) volume (V_{rbc}) are calculated from total blood volume through haematocrit (htc) (pu) as $V_{rbc} = htc \cdot V_b$ and $V_p = V_b - V_{rbc}$. Water, neutral lipids and phospholipid fractions for each tissue are W_f, nL_f and phL_f, respectively. These fractions are not defined for blood, given this tissue was separated into plasma and RBCs, which are considered homogeneous

The venous perfusion blood flow rates for various tissues were proportional to BW, according to the equation $Q_i = q_i \cdot \mathrm{BW}$ (ml/min), for the values q_i as shown in Table 1.

TAC binding is assumed to be a reversible quasi-static process with a single family of binding sites (Toutain and Bousquet-Melou, 2002; Copeland, 2000), according to the following relationship between bound concentration C_b and unbound concentration C_u:

$$C_b = \frac{B_{max} C_u}{K_d + C_u} \tag{3}$$

In short, saturable reversible binding is assumed according to equation (3) for RBCs; linear binding for plasma is $fu_p = C_{up}/C_p$, in which C_p is the total plasma concentration and C_{up} is the unbound plasma concentration. Linear binding for the non-homogenous tissue phase of the remaining organs is $fu_t = C_{ut}/C_t$, in which C_{ut} is the unbound tissue concentration and C_t is the total tissue concentration. The value of fu_t is calculated according to the Langmuir approximation given by equation 4.21 of (Copeland, 2000), as follows:

$$fu_t = \frac{1}{1 + nK_d^{-1} C_{prt}} = \frac{1}{1 + nK_d^{-1} C_{prp} PTP}\,, \tag{4}$$

in which C_{prt} is the tissue binding protein concentration and $PTP = C_{prt}/C_{prp}$ is the ratio of binding tissue / plasma protein concentrations. Given that fu_p verifies the first equality of equation (4) for plasma binding protein concentration C_{prp}, it follows that:

$$fu_p = \frac{1}{1 + nK_d^{-1} C_{prp}}\,, \tag{5}$$

from which the term $nK_d^{-1} C_{prp}$ can be solved and used in equation (4) to obtain fu_t, once the binding tissue / plasma protein ratio PTP is known.

The main binding macromolecules present in plasma and tissue (albumin, globulins, lipoproteins) maintain a tissue plasma ratio from 0.3 to 1 in mammalian tissues, with 0.5 the most frequently reported intermediate value (Poulin and Theil, 2000). Therefore, 0.5 is commonly used as the default value for PTP in PBPK modelling software tools. We used 0.5 for PTP after verifying that the absolute sensitivity of TAC blood concentration to PTP is less than 1, and TAC predictions and interindividual variability were not affected when PTP was perturbed around the value 0.5.

Liver elimination is considered linear and is provided by the intrinsic hepatic clearance per liver volume Cl_{ih} (1/h), which is equal to $Cl_{ih}^{max} \cdot f_h$, in which f_h is the hepatic TAC metabolic activity given by:

$$f_h = f_{m3A5} \cdot f_{a3A5} + f_{m3A4} \cdot f_{a3A4} \cdot (1 - \mathrm{IR3A4}) + f_{moth} \tag{6}$$

The variable f_a is the normalised activity of the hepatic referred cytochrome P450 (CYP), f_m is the fraction of hepatic clearance due to the pathway (CYP3A4/5 or others) and IR3A4 is the inhibition ratio of CYP3A4 activity due to other compounds. The usual values of fractional pathways were taken as $fm_{3A4} = 0.25$, $fm_{3A5} = 0.40$ and $fm_{oth} = 0.35$ (Gérard et al., 2014), whereas normalised activities were defined as 1 for the kidney transplant recipients in our study. Finally, IR3A4 was set at 0 according to the full pharmacological prescription of our study patients.

Assuming that $LibT$ depends mainly on the drug release time, its value for Prograf was calculated using in vitro (simulated gastric fluid, pH 1.2) data for immediate release TAC formulation given by Shin et al. (2018). These data indicate that around 90% (0.9 pu) of the TAC mass is released 1.5 hours after administration . Solving $LibT$ for the zero-order liberation equation (the last equation of Gut system in the Supplementary Material) results in:

$$\frac{1}{LibT} = \frac{0.9}{1.5 \cdot 60} = 0.01\,\mathrm{min}^{-1} \tag{7}$$

Therefore $LibT = 100$ min.

We have verified proper TAC predictions and model robustness during the exploration phase for Prograf with $LibT = 100$ min.

The value of K_{elib} for $LibT = 100$ min was defined to provide an average $F \approx 0.2$ per unit (20%), in agreement with the reported mean value (DrugBank, 2018). This calculation was performed based on a parametric analysis of the PBPK model, using parameters fitted in the initial exploration phase and published values for the rest of model parameters. We obtained $K_{elib}(LibT = 100) = 13.5$ 1/h.

The hematocrit value htc (pu) was calculated based on its relationship with measured haemoglobin concentration Chb (g/dl) (Worrall et al., 2016), equation (8).

$$htc = \frac{Chb}{34} \tag{8}$$

Mathematical model. The model diagram in Figure 2 complements the standard flow diagram of the main text and is built and interpreted in the same manner as the diagram in Figure 1 for the PK model. The detailed scope of each tissue and the entire model are described below by their equations. The main variables and units are defined in the main text.

Heart tissue. This is a flow-limited tissue governed by the following equation (9):

$$V_h \frac{\mathrm{d}C_h}{\mathrm{d}t} = Q_h C_p - Q_h C_h \tag{9}$$

Heart volume flow rate Q_h is the total cardiac output (CO).

Blood system. We modelled Blood using a membrane-limited tissue approach, with plasma and RBC compartments. The approximate distribution of TAC is 95% in RBCs and 5% in plasma, with linear and strong binding in plasma ($fu_p \approx 0.012$)(Nagase et al., 1994). Binding in RBC is extensive and saturable with maximum binding capacity B_{max} and dissociation constant K_d (see main text) (Jusko et al., 1995).

The TAC distribution between RBC and plasma can be written by means of the following equation:

$$0.05 \cdot V_{rbc} C_{rbc} = C_p (V_b - V_{rbc}) \Rightarrow 0.05 C_{rbc} = C_p \cdot (\frac{1}{htc} - 1)\,, \qquad (10)$$

in which C_{rbc} is the RBC TAC concentration.

The transfer of TAC across the RBC barrier is a complex issue, and its active mechanisms are not completely understood. However, a discussion of these mechanisms exceeds the objectives of this model (Tron et al., 2019; W et al., 1993)3. The mechanisms result in lower TAC transport from RBCs to plasma, which is addressed by non-symmetric mass flow rate transport RBC-plasma coefficients, which account for the TAC distribution data (Nagase et al., 1994).

K_{pRBC} and K_{RBCp} (ml/min) denotes the mass flow rate transport clearances from plasma to RBC and vice versa. If the net RBC - plasma TAC flow rate is zero (RBC-plasma TAC fast-equilibrium) then $K_{pRBC} C_{up} = K_{RBCp} C_{urbc}$. The concentrations refer to unbound values in plasma and RBCs. A gross estimate of the mass transport K_{pRBC} and K_{RBCp} relationship can be obtained approximating the previous equation with the following equation:

$$K_{pRBC} C_p = K_{RBCp} C_{rbc} \Rightarrow \frac{K_{pRBC}}{K_{RBCp}} = (\frac{1}{htc} - 1)\frac{1}{0.05} \underbrace{= 24.4}_{htc=0.45}\,, \qquad (11)$$

in which hematocrit has been calculated as the mean value 0.45 pu (45 %).

Fast-equilibrium RBC-plasma is achieved through a sufficiently high value of K_{RBCp}. We set $K_{RBCp} = 10^4$ ml/min, resulting in $K_{pRBC} = 24.4 \cdot 10^4$ ml/min. Preliminary fitting of population PBPK model variables and a sensitivity analysis confirmed the accuracy of the TAC predictions and the robustness of the model to K_{RBCp} and K_{pRBC} perturbations.

TAC concentration in plasma is provided by the following equations:

$$\begin{aligned} C_{up} &= fu_p \cdot C_p \\ V_p \frac{\mathrm{d}C_p}{\mathrm{d}t} &= \sum_i Q_i C_{vi} - Q_f C_p + K_{RBCp} C_{urbc} - K_{pRBC} C_{up}\,, \end{aligned} \qquad (12)$$

in which i takes values f (fat), l (liver), k (kidney) and o (others).

TAC concentration in RBCs is given by the following equations:

$$\begin{aligned} C_{brbc} &= \frac{B_{max} C_{urbc}}{K_d + C_{urbc}} \\ C_{rbc} &= C_{brbc} + C_{urbc} \\ V_{rbc} \frac{\mathrm{d}C_{rbc}}{\mathrm{d}t} &= -K_{RBCp} C_{uRBC} + K_{pRBC} C_{up} \,, \end{aligned} \tag{13}$$

where C_{brbc} is the RBC TAC bound concentration.

Whole blood TAC concentration is calculated with the following equation:

$$C_b = \frac{C_p V_p + C_{rbc} V_{rbc}}{V_p + V_{rbc}} \tag{14}$$

Measurements of TAC concentrations in our clinical study are referred to whole blood; therefore, C_b is the model output used in the parametric fitting.

Fat tissue. This is a flow-limited tissue with a partition ratio R_f and linear binding. TAC tissue concentration is provided by the following equations:

$$\begin{aligned} C_{uf} &= R_f \cdot C_{uvf} \\ C_{uvf} &= fu_p \cdot C_{vf} \\ C_{uf} &= fu_t \cdot C_f \\ V_f \frac{\mathrm{d}C_f}{\mathrm{d}t} &= Q_f C_h - Q_f C_{vf} \end{aligned} \tag{15}$$

Variables C_{uf} and C_{uvf} are the unbound fat tissue and unbound venous fat tissue concentrations. Variables C_f and C_{vf} are the total fat tissue and total venous fat tissue concentration. Equations (15) gives unbound and total concentrations in fat and venous fat phases over time.

We should note the use of the tissue-venous partition ratio R_t, which relates to phase unbound concentrations, unlike the partition coefficient R_{tt} used in other studies, which relates to total concentrations. R_{tt} and R_t are related through the following equation:

$$R_{tt} = \frac{C}{C_p} = \frac{C_u}{C_{up}} \frac{fu_p}{fu_t} = R_t \cdot \frac{fu_p}{fu_t} \,, \tag{16}$$

in which fu_p and fu_t are the unbound plasma fraction and unbound tissue fraction, respectively, described and calculated in the main text.

Liver tissue. Liver tissue is a flow-limited tissue with a partition ratio R_l, linear binding and hepatic clearance, using the following equations:

$$\begin{aligned} C_{ul} &= R_l \cdot C_{uvl} \\ C_{uvl} &= fu_p \cdot C_{vl} \\ C_{ul} &= fu_t \cdot C_l \\ V_l \frac{\mathrm{d}C_l}{\mathrm{d}t} &= (Q_l - Q_g) C_h + Q_g C_{vg} - Q_l C_{vl} - V_l \cdot Cl_{ihchr}(t) C_{ul} \end{aligned} \tag{17}$$

Concentrations follow the same designation as fat (see main text), replacing subindex f with l (subindex g is used for the gut venous blood flow rate). Clearance is chronomodulated as described in the main text.

Kidney tissue. Kidney tissue is a flow-limited tissue with a partition ratio R_k and linear binding. TAC tissue concentration is provided by the following equations:

$$\begin{aligned} C_{uk} &= R_k \cdot C_{uvk} \\ C_{uvk} &= fu_p \cdot C_{vk} \\ C_{uk} &= fu_t \cdot C_k \\ V_k \frac{\mathrm{d}C_k}{\mathrm{d}t} &= Q_k C_h - Q_k C_{vk} \end{aligned} \tag{18}$$

Variables follow the same designation used in previous tissues.

Others tissue. This tissue combines the remaining body tissues not explicitly considered. It is considered a flow-limited tissue with a partition ratio R_o (see main text) and linear binding. TAC concentrations are solved using the following equations:

$$\begin{aligned} C_{uo} &= R_o \cdot C_{uvo} \\ C_{uvo} &= fu_p \cdot C_{vo} \\ C_{uo} &= fu_t \cdot C_o \\ V_o \frac{\mathrm{d}C_o}{\mathrm{d}t} &= Q_o C_h - Q_o C_{vo} \end{aligned} \tag{19}$$

Gut system. This system consists of the gut lumen, where TAC is administered orally and absorbed by gut tissue through a one-order absorption chronomodulated process. The system is described by the following equations:

$$\begin{aligned} C_{ug} &= R_g \cdot C_{uvg} \\ C_{uvg} &= fu_p \cdot C_{vg} \\ C_{ug} &= fu_t \cdot C_g \\ V_g \frac{\mathrm{d}C_g}{\mathrm{d}t} &= Q_g C_h - Q_g C_{vg} + K_{abschr}(t) M_{gl} \\ \frac{\mathrm{d}M_{gl}}{\mathrm{d}t} &= \frac{1}{LibT} M_f - K_{abschr}(t) M_{gl} - K_{elib}(LibT) M_{gl} \\ \frac{\mathrm{d}M_f}{\mathrm{d}t} &= D_f - \frac{1}{LibT} M_f \end{aligned} \tag{20}$$

TAC mass in Gut lumen and oral form, M_{gl} and M_f, are additional variables to those defined in the main text.

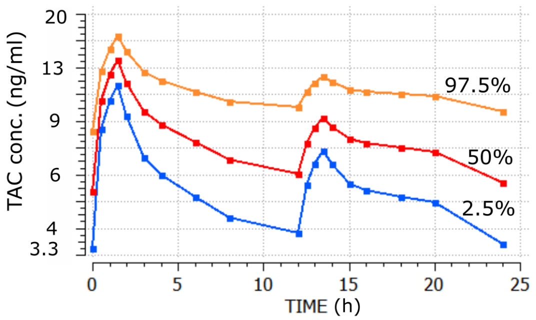

Figure 3: 2.5%, 50% and 97.5% percentiles of TAC blood concentration (ng/ml) obtained with 100 Monte Carlo simulations.

Monte Carlo exploratory analysis of unbound plasma fraction. As described in the main text, the value of fu_p (pu) was set to the mean value 0.012 pu (Zahir et al., 2001).

We performed a Monte Carlo study to verify the robustness of the model with a total of 100 simulations: 10 patients with parameters and dispersions obtained in a preliminary population fitting of the model and 10 fu_p values normally distributed (mean = 0.012, standard deviation = 0.006).

Fig. 3 shows that the TAC blood concentration profiles for the indicated percentiles maintain similar temporal dynamics. The trough TAC concentration C_0 varied from 3.3 to 8.2 ng/ml for the morning dose and from 4.0 to 9.9 ng/ml for the evening dose.

Adaptive modelling techniques

This section details all the boundary equations used with the adaptive Bayesian and weighted least squares (WLS) techniques presented in the main text. Decision variables in the Bayesian technique are based on the population variables selected in the population model fitting. Additional model variables are added in the adaptive WLS technique.

Adaptive Bayesian Optimisation. Model adaptation was focused on correcting blood TAC prediction errors during the second monitoring day.

Boundary equations for adaptive Bayesian optimisation in the PK model are as follows:

$$\begin{gathered}
-2 \leq \Delta\eta_{Cl} \leq 2 \\
-2 \leq \Delta\eta_{Ka} \leq 2 \\
-2 \leq \Delta\eta_{MTT} \leq 2 \\
-0.1\min(\eta_{r_{chr0}}, 0.01) \leq \Delta\eta_{r_{chr}} \leq 0.1\min(\eta_{r_{chr0}}, 0.01) \\
-0.01\min(\eta_{t_{chr0}}, 0.01) \leq \Delta\eta_{t_{chr}} \leq 0.01\min(\eta_{t_{chr0}}, 0.01)
\end{gathered} \tag{21}$$

The value $\eta_{\square 0}$ refers to the value of η (interindividual variation) at the fitting state prior to the adaptation. Incremental bounds and weights are defined to restrict the change in Bayesian model parameters according to the new information. The circadian modulation factor r_{chr} and temporal phase t_{chr} are strongly limited during the adaptation process. These boundaries were evaluated during preliminary explorations of the adaptive phase, and the results confirmed that final optimised variables remained inside the boundary range, with proper convergence of the numerical method. The same methodological strategy was used with the other boundary equations

Boundary equations for adaptive Bayesian optimisation in the PBPK model are as follows:

$$\begin{gathered} -2 \leq \Delta\eta_{Cl_{ih}} \leq 2 \\ -2 \leq \Delta\eta_{B_{max}} \leq 2 \\ -2 \leq \Delta\eta_{K_d} \leq 2 \\ -0.1\min(\eta_{r_{chr0}}, 0.01) \leq \Delta\eta_{r_{chr}} \leq 0.1\min(\eta_{r_{chr0}}, 0.01) \\ -0.01\min(\eta_{t_{chr0}}, 0.01) \leq \Delta\eta_{t_{chr}} \leq 0.01\min(\eta_{t_{chr0}}, 0.01) \end{gathered} \tag{22}$$

Adaptive WLS optimization. This technique was used in two patient scenarios. The weight of the objective function was set to 1 ($W = 1$).

- First scenario. Model adaptation attempts to correct only the trough concentration at the beginning of the second monitoring day.
- Second scenario. Model adaptation attempts to correct the prediction errors during the second monitoring day.

Only one decision variable was defined for the first scenario. This variable was MTT for the PK model and $LibT$ for the PBPK model, with boundary equations as follows:

$$-2 \leq \Delta\eta_{MTT} \leq 2 \tag{23}$$

$$10 \leq LibT \leq 3700 \tag{24}$$

Additional model parameters are added in the second scenario. In the PK model, decision variables and bounds are similar to the incremental Bayesian method, except for the addition of FDT to facilitate the drug formulation conversion. The variable t_{chr} was removed here due to its negligible influence.

$$\begin{gathered} -2 \leq \Delta\eta_{Cl} \leq 2 \\ -2 \leq \Delta\eta_{Ka} \leq 2 \\ -2 \leq \Delta\eta_{MTT} \leq 2 \\ -0.1\min(\eta_{r_{chr0}}, 0.01) \leq \Delta\eta_{r_{chr}} \leq 0.1\min(\eta_{r_{chr0}}, 0.01) \\ -0.08 \leq \Delta FDT \leq 5 \end{gathered} \tag{25}$$

The low incremental limit for FDT ensures that this variable will be greater than 0.0833-0.08 hours (0.2 min), in which 0.0833 h (5 min) was the value set at Prograf fitting.

Decision variables and bounds for PBPK are defined similarly. That is, clearance and absorption parameters, together with drug liberation time, are allowed to change due to the new drug formulation. The negative incremental limit of K_{abs} guarantees that the final value is greater than zero.

$$\begin{gathered} -2 \leq \Delta\eta_{Cl_{ih}} \leq 2 \\ -4.25 \leq \Delta K_{abs} \leq 9 \\ -0.1\min(\eta_{r_{chr0}}, 0.01) \leq \Delta\eta_{r_{chr}} \leq 0.1\min(\eta_{r_{chr0}}, 0.01) \\ 10 \leq LibT \leq 3700 \end{gathered} \tag{26}$$

Model analysis

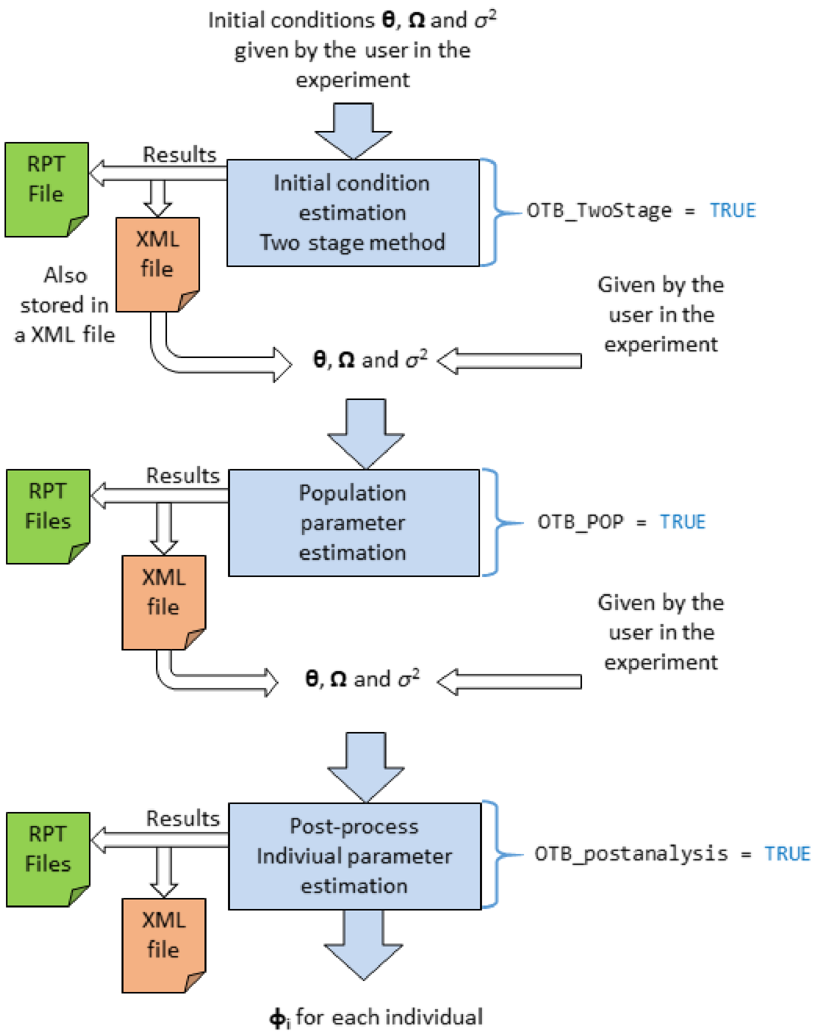

Figure 4: Stages of model's parameter estimation procedures.

Population fitting. Fig. 4 shows the sequence of stages followed by a general population parameter estimation study in PhysPK. All of them are optional, and their execution depends on the value of the associated flag (OTB_TwoStage, OTB_POP, OTB_postanalysis). Results of each stage have been saved in a XML file that can be read by the following step (automatic mode)

The *first stage* performs a preliminary analysis before executing the final population estimation stage, which is not based on the likelihood maximisation

technique but on least squares minimisation. This old population parameter estimation method gives gross estimates for central parameters θ and population variances. The central parameter is related to the individual parameter ϕ through the interindividual dispersion model, which is given by the following equation:

$$\phi = \theta \cdot e^{\eta}\,, \tag{27}$$

in which η is the interindividual dispersion.

Taken advantage of the low CPU demand of this computational step, we used it for three main tasks:

- Computing initial guesses for central parameters, *theta*, covariance matrix, $\mathbf{\Omega}$, and residual variances.
- Analysing potential relationships among individual ϕ and covariate candidates.
- Modifying and evaluating various population variable sets, their population variability and the model robustness for those selections.

We followed this technique during the initial exploration of our models. The first simulations were executed without covariate regression models. The first individual parameter fitting based on minimisation of the least-square residual error was performed initially for randomly selected patients. Plots with selected magnitudes gave us an initial idea of the model's general behaviour for the chosen parameter values.

Subsequent executions of the two-stage (TS) method gave us initial guesses for θ and variances, as well as suggestions for nonvariable population parameters.

Covariate candidates were analysed, seeking correlations between potential covariates and the values of each ϕ estimated for each patient. Selected covariate relationships were subsequently added to the preliminary TAC model. We then performed parametric analyses modifying covariate values and model correlation coefficients for the operational point defined by the previous estimated θ values. Correlation models that gave robust numerical values closest to the known range of AUC_{24} were selected. We also evaluated other model output variables such as F to ensure that their range was correct.

Monte Carlo studies with a few simulations (100) with selected covariate correlations were executed to confirm the range of AUC_{24} with 2.5% and 97.5% percentiles.

A final execution of the TS method with selected covariates and regression models was performed. Coefficients of correlation models were assigned various values with the goal of selecting one of them as an initial guess for the next stage of parameter estimation. Correlations between population variables might have been detected in this step (non-diagonal Ω). However, they were not found, in agreement with other authors (Andreu et al., 2015).

The initial exploration ended with the computation of the dynamic sensitivity of blood TAC concentration (log-transformed) with respect to the parameters of the model, to verify the stability and robustness of the model structure.

The normalised sensitivity of $y(t)$ with respect to the $\phi(t)$ parameter is defined as follows:

$$S_y^{\phi}(t) = \frac{\phi(t)}{y(t)} \frac{\partial y(t)}{\partial \phi(t)}\,, \tag{28}$$

in which y(t) is taken as TAC concentration (log-transformed) at time t and $\phi(t)$ is the model's parameter with respect the sensitivity is computed. Model parameters can be time-dependent, as occurs with parameters that have circadian modulation. In general, the sensitivity $S_y^{\phi}(t)$ varies with time. Absolute values of sensitivity much larger than 1 can induce numerical stability problems. On the other hand, values very close to zero at any time instant t suggest that the measurements of y at t will have scarce influence on the fitting of ϕ. Typically, $S_y^{\phi}(t)$ grows just after a new drug administration, indicating the temporal range when measurements have more influence on the fitting of the ϕ value

The second stage of Figure 4 executes the population model fitting using Likelihood maximisation with additive and proportional residual error models for the measured TAC concentration (log-transformed). The First Order Conditional Estimation (FOCE) (see main text) method was applied to the covariate forward stepwise approach, once the residual error model with better behaviour (lower IIV values and parameter standard errors) was selected. The FOCE-I method did not improve the goodness of estimation (parameter accuracy for either IIV values); thus, FOCE results were selected in our study.

Optimisation mathematical techniques related to likelihood maximisation in PhysPK include the possibility of organising parameters to be estimated in groups, in an iterative procedure controlled by the user. Grouping of parameters was based on differences in the sensitivity analyses and in their physiological or pharmacokinetic meaning. This technique helps prevent local extremes or numerical convergence problems, particularly with gradient-based optimisers. PK model parameters Cl, K_a, MT, V_c, V_p and Cl_d were assigned to group 1, r_{chr} to group 2 and the coefficient of selected covariate correlation to group 3.

With respect to Ω, the variances ω_{Cl}^2, $\omega_{K_a}^2$, and ω_{MT}^2 were assigned to group 1, and $\omega_{r_{chr}}^2$ to group 2. The value of $\omega_{t_{chr}}^2$ was set to 0.01 according to exploration results. An approximate estimation explains this value. Considering Normal distribution and 99.7 % = 1 h, $3\,\omega_{t_{chr}} \leq 1/2$, and thus $\omega_{t_{chr}}^2 < 0.02$.

PBPK model parameters were all assigned to group 1, except for r_{chr}, which was assigned to group 2. The parameter t_{chr} was addressed as it was in the PK model. Concerning the Ω matrix, all variances were in group 1, except for $\omega_{r_{chr}}^2$, which was in group 2.

Once the parameter population distribution was known (mean and dispersion), the model was customised to each patient at the third stage of Figure 4, maximising its likelihood through the Bayesian theorem and the prior distribution (Kelman et al., 1982). As a result, the set of η values that gave the set of model parameters ϕ for each patient was obtained

The initial values of the model state variables are customised for each patient in the 3 stages of Figure 4, using a PhysPK technique called *conditioned simulation*. This technique performs simulation loops based on the drug admin-

istrations defined in the data set file for a particular patient, until a user-defined condition is fulfilled. Common system conditions are the steady state of the system or the achievement of a particular exposition in a compartment.

After various evaluations, the condition selected was the number of drug cycles (days) prior to the first admission to hospital, where TAC measurements were acquired. Although the patients followed the controlled Prograf administration 6 days prior to admission, it was verified that 2 days were sufficient to achieve the steady state. Therefore, only 2 simulation loops were selected as the condition to be fulfilled, to reduce the computing load. As a consequence, the absolute simulation time at hospital admission started at 48 hours. This time value may be considered a reference.

Adaptive procedures evaluation. Figure 5 presents the 2 procedures designed to evaluate the adaptive techniques and models of the study. Plots are based on the PBPK model and a randomly selected patient (number 5) was taken as an example. The fundamental details of the procedures are described in the main text

The top left plot shows the evolution of TAC concentrations (log-transformed) from the beginning of day 1 (first admission day to hospital) to the end of day 8 (hospital re-admission), both according to the PBPK model simulation (orange, 1 to 8 days) and blood TAC measurements (violet, 1 and 8 days). The first sampling absolute time at day 1 occurs at time = 48 hours, as a common reference for all patients indicated in previous Section. Accordingly, the last measurement of blood TAC taken at the end of the hospital re-admission occurs at time = 240 h ($48 + 8 \cdot 24$). This first plot shows that model predictions a week after TAC formulation conversion (beginning of day 2) from Prograf to Advagraf are inaccurate.

Procedure 1 (bottom block) is composed of a first adaptation using the adaptive WLS, to correct the predicted trough value just before the Advagraf administration at day 8 (first measurement of TAC marked in red colour). The WLS technique calculates the change in MTT in the PK model or $LibT$ in the PBPK model, as described in the theoretical section of the main text. The bottom left plot shows the predicted corrected TAC concentration and the TAC measurement (C0, time = 220 h) after model adaptation. That parametric adjustment is applied at time = $48+24 = 72$ h, that is, when the TAC formulation conversion occurs (beginning of day 2). The simulated TAC concentration as a function of time is presented from the beginning of day 2 until beginning of day 8 (72 to 220 h).

A second stage in procedure 1 applies adaptive WLS and Bayesian techniques after the first adaptation. Adaptive methods start from a model with an accurate prediction of TAC concentration at the beginning of the second monitoring day (day 8) and are based on TAC measurements acquired during that day. Any relevant change in the model's parameters at this second stage of procedure 1 suggests that the intrapatient variability is significant. The 2 bottom plots at the right show the corrected TAC concentration predictions

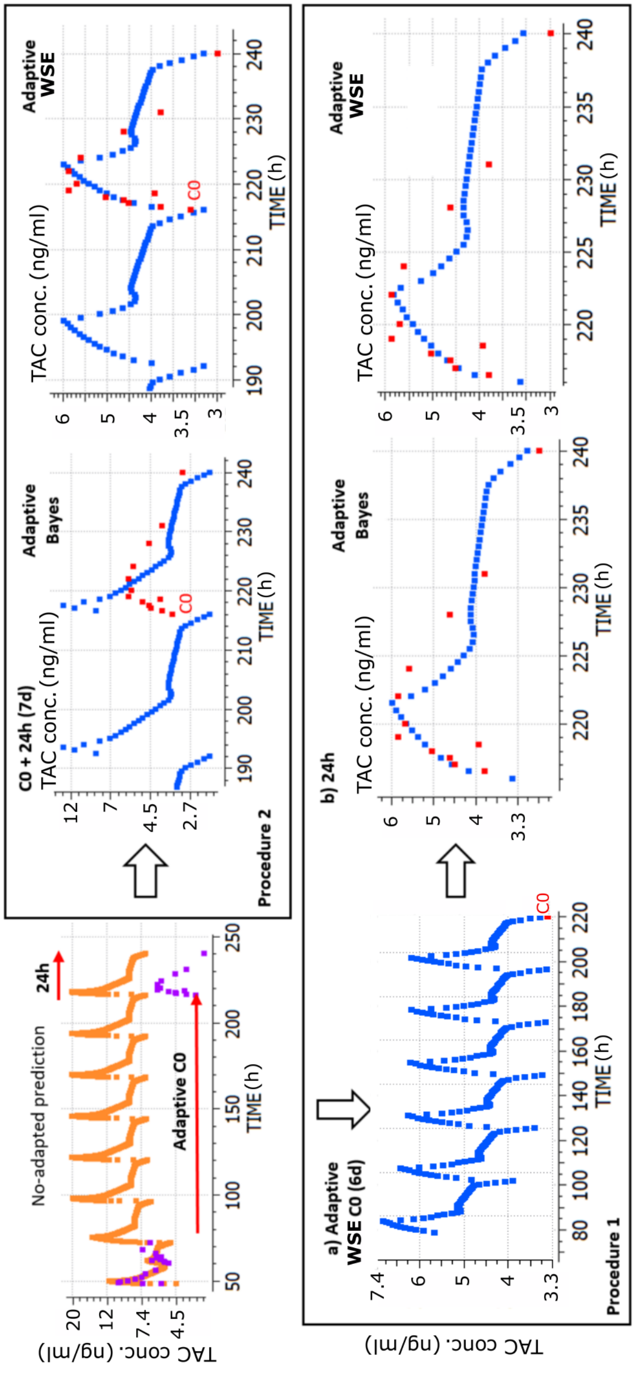

Figure 5: Procedures used for evaluation of adaptive models (see text for details).

versus the actual TAC measurements during the second monitoring day (24 h), for adaptive Bayesian and WLS techniques.

Procedure 2 applies adaptive Bayesian and WLS techniques to correct deviations in TAC predictions during day 8 in a unique stage. The parametric adjustment is applied at time = 72 h (beginning of day 2). This evaluation procedure is compared to the first procedure in the main text. The 2 upper plots at the right of the Figure 5 show the corrected TAC concentration predictions versus actual TAC measurements during the second monitoring day, for adaptive Bayesian and WLS techniques

Results

Fitting and evaluation of PK model (Prograf data)

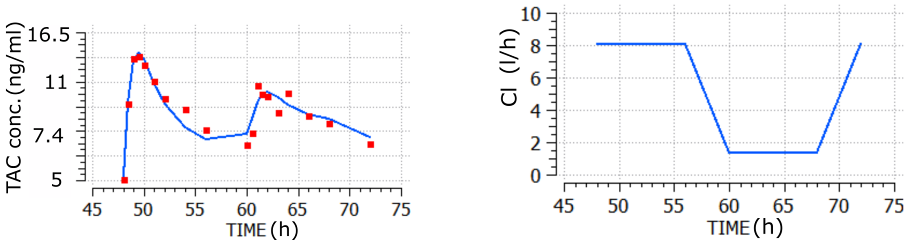

(a) Central TAC concentration (solid) in PK model versus TAC measurements (dots) in (ng/ml) vs time (h)

(b) Circadian modulation of TAC central clearance in the PK model (Cl in l/h vs time in h).

Figure 6: Fitted model with Prograf administration for randomly selected Patient 1 during exploration phase.

Exploration phase. Fig. 6 shows the TAC concentrations (log-transformed) and the temporal variation of systemic clearance Cl due to the circadian rhythm ($Cl_{chr}(t)$) for the randomly selected Patient 1. Plots start at the reference time t=48 h, according to the conditioned simulation described in the Methods section.

Table 2: θ and IIV variances (η variances) estimated for the PK model by means of the TS technique during the exploratory analysis. The exponential correlations model coefficient was 0.1.

θ_{Cl} (l/h)	5.1	ω^2_{Cl}	3.02
θ_{Ka} (ml/h)	34.9	ω^2_{Ka}	1.29
θ_{MTT} (h)	1.9	ω^2_{MTT}	0.47
$\theta_{r_{chr}}(pu)$	0.25	$\omega^2_{r_{chr}}$	0.72
$\theta_{t_{chr}}$ (h)	0.34	–	
θ_{CLd} (l/h)	54.6	–	
θ_{Vc} (l)	15.0	–	
θ_{Vp} (l)	182.5	–	

Additive residual variance $\omega_r^2 = 0.032$.

The PK model was fitted for this patient by the minimisation of least-squared errors between central and blood TAC concentration (log-transformed). The values of the fitted parameters were $Cl = 8.1$ l/h, $K_a = 20.8$ ml/h, $MTT = 1.73$ h, $Cl_d = 49.7$ l/h, $V_c = 11.6$ l, $V_p = 464.7$ l, $r_{chr} = 0.17$ and $t_{chr} = 0.49$ h. TAC exposition (AUC) 24 hours after drug administration as computed by the PK model was $\text{AUC}_{24} = 205.9$ ng/ml$\cdot$h, whereas the log-trapezoidal value based on

the measurements (NCA) was $\mathrm{AUC}_{24} = 207.8$ ng/ml·h. Bioavailability as given by the model was $F = 18.0\%$. RMSE between central TAC concentrations and blood measurements (log-transformed) was $\mathrm{RMSE}_c = 0.034$.

The least-squared error minimisation was subsequently applied to the full population, by means of the TS technique, to obtain gross estimates of θ_i and η-variances ω_i^2 of the PK model parameters.

Potential correlations between covariates and guess estimations of model parameters were evaluated to detect significant correlation coefficients according to linear and exponential regression models. Two relationships were selected at this phase, BSA (m^2) - Cl_d (l/h) with the greatest correlation coefficient and Chb (g/dl) - V_p (l) with a smaller correlation coefficient. Plots of covariates candidates can be found in the Appendix.

PK model parametric analyses were executed using the linear and exponential correlations, with BSA and Chb as variable parameters (first analysis) and with correlation coefficients as variable parameters (second analysis), using the population parameters estimated in the TS stage. The model confirmed the robustness with respect to correlations parameters, with AUC_{24} within the population data range. Exponential correlation models for candidate covariates showed more reduced and proper (more similar to clinical data) values of AUC_{24}.

By performing the Monte Carlo method with 100 simulations at this point, using the variances estimated by the TS technique, we confirmed that TAC concentrations and AUC_{24} were consistent with the clinical population data, particularly with the exponential regression models.

We performed a second execution of the TS method with selected covariate regression models and initial values for θ_i and Ω entries from the previous TS execution. Values for the exponential correlation coefficients were taken as 0.1, 0.3 and 0.5. Table 2 shows the results of the TS method for exponential coefficients equal to 0.1, because this value resulted in the lowest interindividual variance. The smallest estimates of variances occurred in the V_c, V_p and Cl_d variables, which were selected as nonvariable population parameters. These parameters were used as initial guesses in the population fitting phase of the PK model with Prograf administration (see main text).

We completed the initial exploration by calculating the normalised sensitivities of the TAC concentrations (log-transformed) to PK model parameters, as shown in Figure reff:PKsens. The results confirmed the model's structural stability. Plot 7d also confirmed that 48 h were sufficient to achieve a near steady-state situation for TAC concentrations.

The parameters presented in Figure 7 are clustered into variable parameters (Fig. 7a), nonvariable parameters (Fig. 7b) and circadian parameters (Fig. 7c). The first group (θ_{Cl}, θ_{Ka} and θ_{MTT}) had the strongest influence on TAC concentrations, whereas the second group had less influence, particularly θ_{Vc}. The parameter $\theta_{t_{chr}}$ exerted a very small influence on TAC concentrations.

The sensitivities of TAC concentrations to the exponential coefficients of the covariate correlation models were also very low. Their behaviour was similar to that of $\theta_{r_{chr}}$ in Figure 7c.

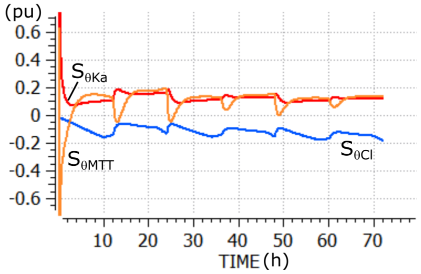

(a) Normalised sensitivities of TAC to θ_{Cl} (blue), θ_{Ka} (red) and θ_{MTT} (orange).

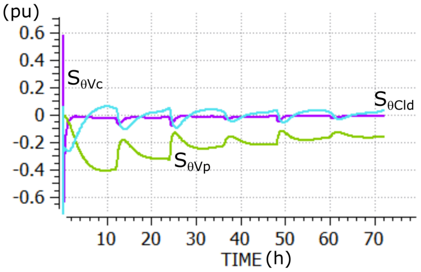

(b) Normalised sensitivities of TAC to θ_{Vc} (purple), θ_{Vp} (green), θ_{CLd} (clear blue).

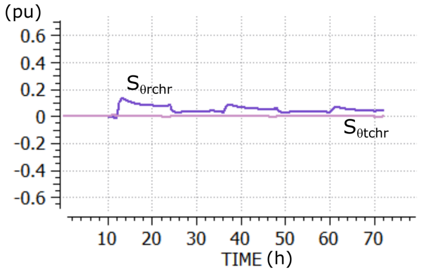

(c) Normalised sensitivities of TAC to $\theta_{r_{chr}}$ (dark violet) and $\theta_{t_{chr}}$ (soft violet).

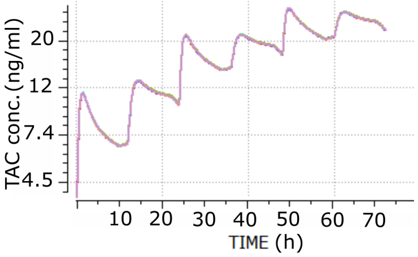

(d) Evolution of log-transform central TAC concentration in the sensitivity study.

Figure 7: Exploratory sensitivity analysis of the log-transform TAC concentration (ng/ml) to the indicated parameters. Time is in hours.

Likelihood-based population fitting Figure 8 presents model TAC predictions, fitted with the Prograf data from our stable paediatric kidney transplant recipients. TAC predictions and measurements were selected for 5 random patients. Two patients were extracted from the top plot and are presented in the bottom plots to clarify the behaviour of the TAC predictions. Patient 10 (Fig. 8b) showed the most common TAC temporal pattern in our population.

Table 3 shows a comparison with parameters obtained by Andreu et al. (2015) model. The MTT parameter from Andreu et al. has been converted through $MTT/3$ to agree with our MTT definition. We modified the K_a from Andreu et al. to $K_a \cdot V_{si} = K_a \cdot 0.13 \cdot 0.475 \cdot 10^3 = K_a \cdot 61.7$ to agree with our K_a definition (see the mathematical development of the PK model). The parameters' values are written as value/BW (kg). The ratio value/BW is presented to facilitate the comparison between models because of differences between adult (Andreu et al.) and paediatric (our PK model) kidney transplant recipients. Comparisons between residual variabilities should consider the different type of error function (proportional in the Andreu et al. model and additive in our PK model). The proportional error variable multiplies the output $y = ln(C)$ in the Andreu et al. model, which in our study has values ranging from 1.1 to 2.3 (blood TAC concentration C in ng/ml, second supplementary file online).

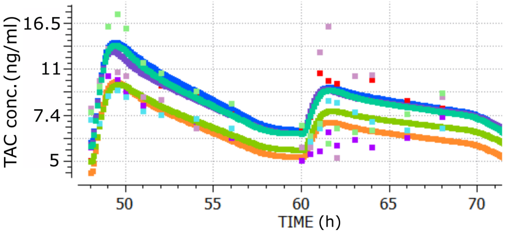

(a) Prograf fitted PK model simulation for Patients 1, 5, 10, 15, and 20.

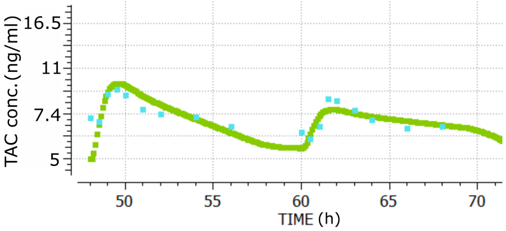

(b) Prograf fitted PK model simulation for Patient 10.

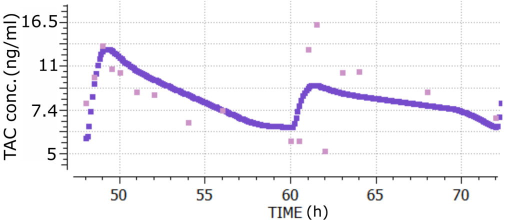

(c) Prograf fitted PK model simulation for Patient 15.

Figure 8: TAC central concentrations (log-transformed) from PK model simulations and clinical sampling values (ng/ml) vs. time (h).

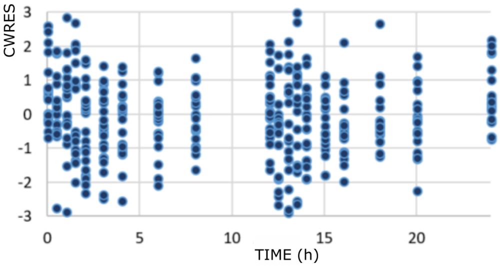

Figure 9: CWRES vs. time after dose for PK model.

Table 3: Prograf PK model estimates vs. Andreu et al. (2015). Values in brackets are scaled by the mean BW of each population: 42.85 kg (current), 68 kg (Andreu)

**Parameter(Units)**	**PK model**	**Andreu**
θ_{Cl} (L/h)	5.41 (0.13)	16.50 (0.24)
θ_{CLd} (L/h)	46.5 (1.09)	35.56 (0.52)
θ_{Vc} (L)	23.0 (0.54)	9.89 (0.14)
θ_{Vp} (L)	39.1 (0.91)	526.03 (7.74)
θ_{Ka} (ml/h)	13.8 (0.32)	29.0 (0.43)
θ_{MTT} (h)	0.72 (0.017)	0.28 (0.004)
$\text{IIV}_{Cl}^{\S}$ (%)	31	39
IIV_{Ka} (%)	57	35
IIV_{MTT} (%)	22	32
resV (%)	$16^{\ddagger}$	$21^{\dagger}$

§IIV and residual variability (resV) expressed in CV. †Proporcional. ‡Additive.

In terms of the normal distribution assumption for parameters and errors, Figure 9 presents the conditional weighted residuals (CWRES) - time for the fitted TAC PK model. The plot shows the time after diurnal and nocturnal Prograf doses. Although the mean CWRES values deviate slightly from zero at several sampling times, the diagnostic plot indicates that the PK model properly fits the simulated data.

We performed a Monte Carlo study of 600 simulations with the PK model for 3 TAC normalised doses to find a dose value as a reference for paediatric kidney transplant recipients. Table 4 shows the AUC_{24} results. Each simulation was performed for 48 + 24 hours. Therefore, the time that TAC concentration surpasses MTC (20 ng/dl) (t_{MTC}) is referred to as 72 hours.

Table 4: Values of AUC_{24} for 600 simulations with 3 different doses per kg of body weight with the PK model.

**Dose mg/kg**	**AUC_{24} ng/ml · h mean (SD)**	**t_{MTC} h mean (SD)**	**F % mean (SD)**
0.025	165.5 (107.3)	0.4 (1.9)	20.9 (8.9)
0.042	271.4 (196.7)	6.5 (15.4)	20.1 (8.7)
0.05	343.7 (231.2)	11.3 (19.3)	21.6 (9.9)

Fitting and evaluation of PBPK model (Prograf data)

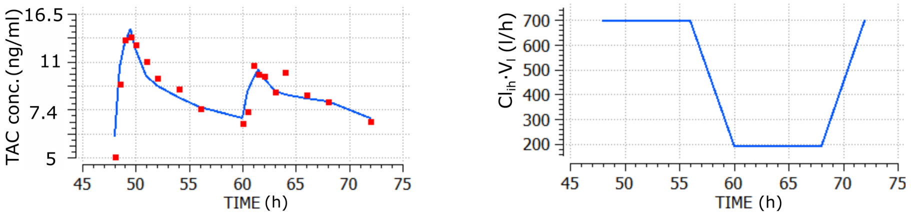

(a) TAC blood concentration in the PBPK model (solid) vs. blood TAC measurements (dots) (ng/ml) vs. time (h)

(b) Circadian modulation of TAC hepatic intrinsic clearance in PBPK model ($Cl_{ih} \cdot V_l$ in L/h vs. time in h).

Figure 10: Fitted PBPK model for randomly selected Patient 1 during the exploration phase.

Initial exploration. Figure 10 shows a TAC concentration (log-transformed) and circadian modulation of intrinsic hepatic clearance $Cl_{ih} \cdot V_l$ (l/h) predicted with the PBPK model for Patient 1. The model was fitted by minimising the least-squared errors between simulated and blood measurements of TAC concentrations. Fitted parameters were $Cl_{ih} = 591.1$ 1/h, $B_{max} = 103.3\mu$g/L, $K_d = 31.2\mu$g/L, $r_{chr} = 0.28$ per unit (pu) and $t_{chr} = 0.107$ h. TAC exposition was $\text{AUC}_{24} = 204.0$ ng/ml $\cdot$ h (PBPK model), and $\text{AUC}_{24} = 207.8$ ng/ml $\cdot$ h (NCA log-trapezoidal). Bioavailability as computed by the model was $F = 16.7$ %. The RMSE between the model blood TAC concentrations and blood measurements (log-transformed) was $\text{RMSE}_c = 0.044$ for this patient.

We applied the least-squared error minimisation technique (TS), parametric analysis and exploratory Monte Carlo in the same manner as in the PK model. However, we did not find additional covariates to those used in the mechanistic equations (see Model development).

Table 5: θ and IIV (η variances) estimated for the PBPK model by the TS technique during exploratory analysis.

θ_{CLih} (1/h)	719.2	ω^2_{CLih}	0.13
θ_{Bmax} (ng/ml)	178.0	ω^2_{Bmax}	0.99
θ_{Kd} (ng/ml)	36.8	ω^2_{Kd}	0.07
$\theta_{r_{chr}}$ (pu)	0.11	$\omega^2_{r_{chr}}$	1.67
$\theta_{t_{chr}}$ (h)	0.07	–	

Estimated additive residual variance $\omega_r^2 = 0.031$.

The final execution of the TS technique for the PBPK model resulted in the estimated mean θ_i parameters and gross estimated Ω entries shown in Table 5. The estimated covariances between the parameters were negligible, and thus Ω was set as a diagonal matrix. Values for the previous table were used as

initial guesses in the population-fitting phase of the PBPK model with Prograf administration.

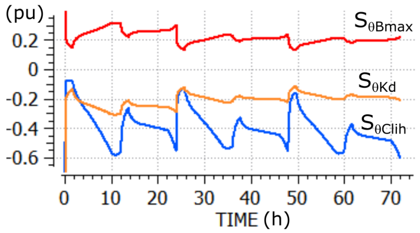

(a) Normalised sensitivities of TAC to θ_{CLih} (blue), θ_{Bmax} (red) and θ_{Kd} (orange) with $LibT = 100$ min.

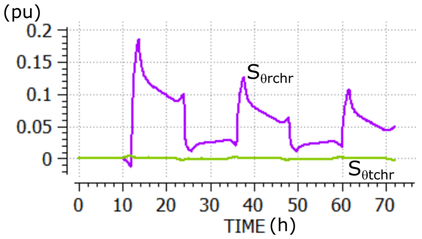

(b) Normalised sensitivities TAC to $\theta_{r_{chr}}$ (violet) and $\theta_{t_{chr}}$ (green) with $LibT = 100$ min.

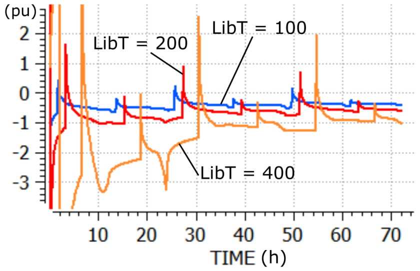

(c) Normalised sensitivities of TAC to $LibT$ with $LibT = 100$ min (blue), $LibT = 200$ min (red), and $LibT = 400$ min (orange).

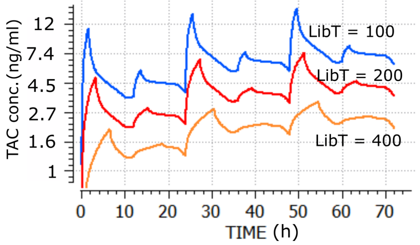

(d) Log-transform blood TAC concentration for $LibT = 100$ min (blue), $LibT = 200$ min (red) and $LibT = 400$ min(orange).

Figure 11: PBPK model exploratory sensitivity of log-transform blood TAC concentration (ng/ml) to the indicated parameters. Time in hours.

To finalise the exploration stage, we conducted a dynamic normalised sensitivity study with the population parameters defined by values found through the TS technique. Other model parameters were defined by their physiological or average representative values (e.g., body weight).

Figure 11 shows the sensitivity of TAC concentrations (log-transformed) with respect to the mean population parameters of PBPK, clustered in variable parameters (Fig. 11a), circadian parameters (Fig. 11b) and LibT (Fig. 11c). The plots confirmed the structural stability of the PBPK model. Plot 11d showed that steady-state TAC concentrations are reached at approximately 48 h in the PBPK model

TAC sensitivities are greater at time points after and near Prograf administrations. Therefore, measurements obtained at those time points have greater influence than others during the model fitting.

The parameter θ_{Clih} has a greater influence on TAC blood concentrations than the RBC binding parameters. The parameter $\theta_{t_{chr}}$ scarcely affects TAC concentrations, as occurs in the PK model.

The $LibT$ parameter was included in the sensitivity study, even though it was not defined as a population fitting variable. This variable is related to the TAC formulation and is therefore relevant for understanding the behaviour of the PBPK model when $LibT$ changes. As shown, the average TAC absolute sensitivity to $LibT$ increases with the value of $LibT$, in addition to presenting abrupt behaviour close to TAC administrations. However, the absolute values for sensitivity are moderated and do not affect the model's stability.

Plot 11d shows that maximum TAC concentrations ($C_{max}(t_{max})$) decrease and that t_{max} increases when $LibT$ increases. This drug behaviour is expected when associated with a prolonged-release formulation.

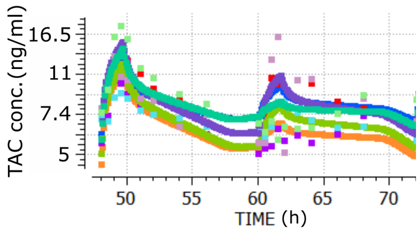

(a) Prograf fitted PBPK model simulations for Patients 1, 5, 10, 15, and 20.

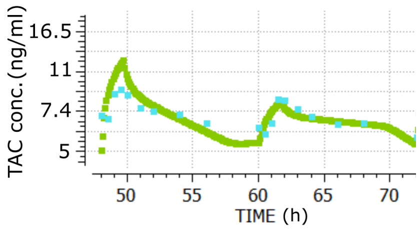

(b) Prograf fitted PBPK model simulation for Patient 10.

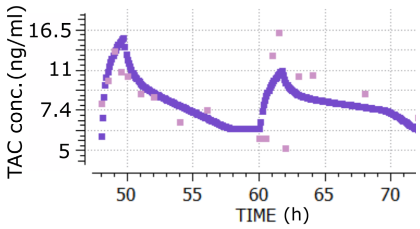

(c) Prograf fitted PBPK model simulation for Patient 15.

Figure 12: Blood TAC concentration (log-transformed) from the PBPK model simulation and blood measurements (ng/ml) vs. time (h).

Likelihood-based population fitting. Figure 12 shows the blood TAC concentration (log-transformed) predictions of the PBPK model, fitted with Prograf data from our stable paediatric kidney transplant recipients. We selected plots and measurements for 5 random patients.

Patients 10 and 15 were extracted from the top plot and are presented in the bottom plots to clarify the TAC concentration behaviour. Comparisons of PBPK TAC temporal concentration with PK TAC temporal concentrations (Fig. 8b and Fig. 8c) show several slight differences in TAC patterns (dynamics). For example, the PBPK model presents faster changes in TAC slopes (derivative) than in the PK model. The TAC patterns are consistent with the circadian rhythms (Barbarino et al., 2013).

Population parameters estimated for our PBPK model can be compared with other published values. The mean intrinsic hepatic clearance ($Cl_{ih} \cdot V_l$) was 922.4 l/h for a BW of 55 kg. This intrinsic hepatic clearance is much lower than the 10600 l/h calculated by Gérard et al. (2014). However, Gerard et al. determined this parameter with only 6 adult liver transplant recipients and did not consider the circadian modulation effect. In fact, the predictions versus the observed TAC concentrations from the study by Gerard et al. are within the range of 0.33-fold to 3-fold in approximately 90% of the cases. These high TAC deviations could be explained by the fact that the authors' primary intention was to identify the most influential variability factors in TAC trough blood concentrations and not to achieve an accurate predictive model. The predicted intrinsic hepatic clearance from human liver microsomes was 2943.6 l/h, with a range of 1032.9-4851 l/h for a BW of 55 kg (Gertz et al., 2010), which is closer to our estimate.

The estimated mean RBC TAC binding values presented in the main text, $B_{max} = 11$ μg/l and $K_d = 1.6\mu$g/l, can be compared with the mean values for adult liver transplant recipients ($B_{max} = 418$ μg/l and $K_d = 3.8$ μg/l) (Jusko et al., 1995). The discrepancy could be explained by differences in our paediatric population, consistent with the high variability found for these binding parameters for various populations (Jusko et al., 1995).

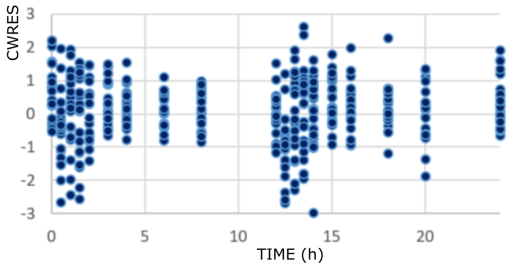

Figure 13: CWRES vs. time after dose for the PBPK model.

Figure 13 presents the CWRES versus time for the fitted TAC PBPK model. The plot shows the time after diurnal and nocturnal Prograf doses. This diagnostic plot indicates that simulated data are properly fitted in the PBPK

model.

Table 6: Values of AUC_{24} for 600 simulations with 3 different doses per kg of body weight with the PBPK model.

**Dose** mg/kg	**AUC_{24}** ng/ml · h mean (SD)	**t_{MTC}** h mean (SD)	**F** % mean (SD)
0.025	115.6 (32.3)	0 (0)	15.5 (1.7)
0.042	177.6 (50.9)	0.005 (0.039)	15.4 (1.8)
0.05	208.9 (58.4)	0.075 (0.30)	15.5 (1.6)

Results from the Monte Carlo study with 600 simulations of the PBPK model for 3 Prograf dose levels administered every 12 h are shown in Table 6. Input model variables such as BW, BSA, haemoglobin, sex, height and age varied according the values found in our paediatric population. Simulations were executed over 72 h to ensure a near steady-state situation at the third day of the simulation. Values of AUC_{24} refer to the previous 24 hours.

PBPK model: Adaptive procedure 1 (Advagraf data).

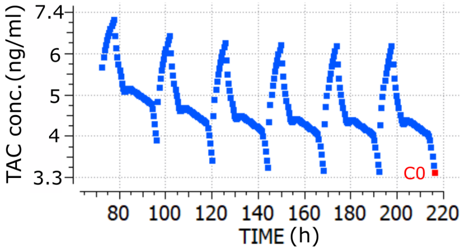

(a) Adapted PBPK model customised to Patient 5.

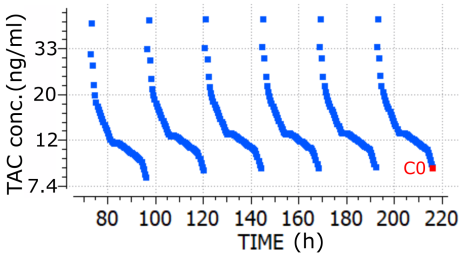

(b) Adapted PBPK model customised to Patient 15.

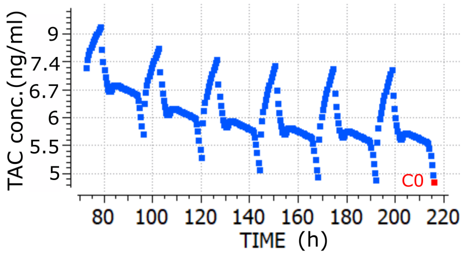

(c) Adapted PBPK model customised to Patient 20.

Figure 14: Adapted PBPK model TAC concentrations (log-transformed) predictions (stage 1 of procedure 1) versus measured blood TAC concentration at the beginning of day 8 (C0). Time in hours.

Figure 14 shows the evolution of the PBPK model for 3 randomly selected patients after adjusting the *LibT* value (stage 1 of procedure 1) to correct the trough TAC concentrations at the beginning of the first hospital re-admission day.

Table 7 shows the adjusted *LibT* values, with the columns presenting *LibT* values at Prograf fitting (day 1) and after executing the WLS adaptive technique (WLS C0). The second block of the table presents the C0 prediction deviation

(Δ C0) before (Prograf) and after (WLS C0) the adaptation.

Table 7: PBPK model corrected (*LibT*) with the WLS adaptive technique to improve trough TAC concentrations at the beginning of day 8. Values for Patients 5, 15 and 20. See text.

**Parameter**	**Prograf**	**WLS C0**
**Patient 5**		
LibT (min)	100	341.4
**Patient 15**		
LibT (min)	100	58.9
**Patient 20**		
LibT (min)	100	393.3
**Metrics**	**Prograf**	**WSE C0**
**Patient 5**		
Δ C0†(ng/ml)	3.61	0.009
**Patient 15**		
Δ C0 (ng/ml)	-2.16	0.093
**Patient 20**		
Δ C0 (ng/ml)	6.47	0.005

† Difference with respect to the measurement.

LibT increased the value of the Advagraf formulation, with the exception of Patient 15. The reduction in *LibT* in that patient was related to the undervaluation of measured C0 by the model before adaptation, in contrast to the other two patients. Blood TAC AUC_{24} was reduced from 155.9 to 110.0 ng/ml · h (Patient 1) and from 206.2 to 140.8 ng/ml · h (patient 20) and increased from 196.4 to 367.4 ng/ml · h for Patient 15, who was the only patient with a reduced *LibT* after PBPK model adaptation.

The predicted blood TAC concentrations versus blood TAC measurements after the Advagraf administration on day 8, after the PBPK model was readjusted in the second stage of procedure 1, are shown for the same 3 patients as Figure 15. The left plots were obtained with the Bayesian adaptive technique, and the right plot was obtained with the WLS adaptive technique. Both adaptive methods achieved an improvement in the objective function (likelihood in Bayesian and least squares in WLS) and thus in TAC predictions during day 8.

Predictions for TAC concentrations for Patient 20 show that the Bayesian adaptive method might have limitations in achieving accurate predictions through the adapted model in cases in which *LibT* requires a significant adjustment.

The values of the model parameters after adaptation appear in Table 8. C0-instant refers to the beginning of re-admission to hospital. Values of parameters at this time point are equal to those set at the Prograf fitting, with the exception of *LibT*. The goodness of fit was better for the adaptive WLS than for the adaptive Bayesian technique, according to the $RMSE_c$ and AUC_{24} values.

This result agrees with the TAC temporal patterns shown in Figure 15. The hepatic intrinsic clearance Cl_{ih} and the absorption constant rate K_{abs} appear to be the parameters that govern the readjustment, whereas those related to

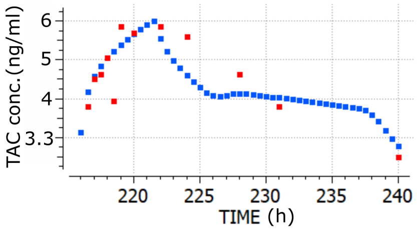

(a) Patient 5 with adaptive Bayesian.

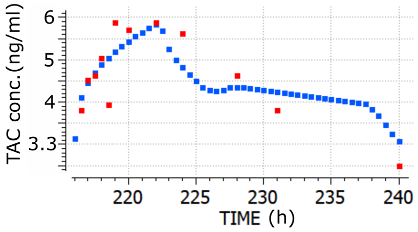

(b) Patient 5 with adaptive WLS.

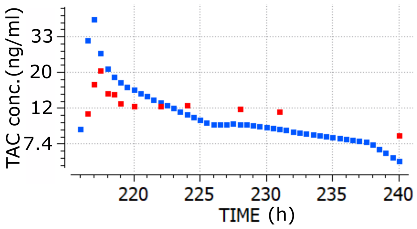

(c) Patient 15 with adaptive Bayesian.

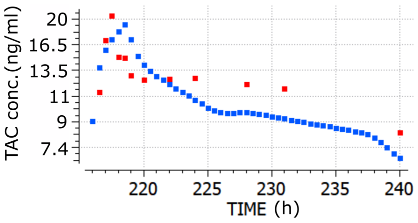

(d) Patient 15 with adaptive WLS.

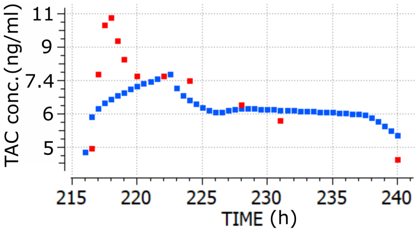

(e) Patient 20 with adaptive Bayesian.

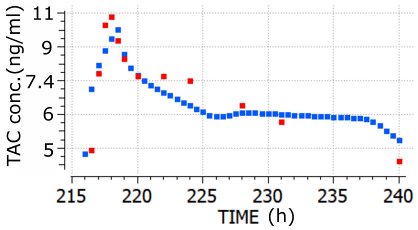

(f) Patient 20 with adaptive WLS.

Figure 15: Predicted blood TAC concentrations (blue) with adapted PBPK model (second adaptation, procedure 1) during the day of readmission compared with blood TAC measurements (red) (log-transformed) for selected patients. Time in hours.

RBC binding B_{max} and K_d, and circadian activity reduction r_{chr}, are scarcely changed.

The r_{chr} parameter is not presented in the entire patient table (see main text) because its variation was very close to zero with both adaptive techniques, and it could be extracted from the decision variables without affecting results.

Table 8: PBPK model parameters at the second adaptation of procedure 1 for Patients 5, 15 and 20. Accuracy metrics: AUC_{24} and $RMSE_c$ (log-transformed).

**Params**[†]	**C0-instant**	**Bayes**	**WSE**
**Patient 5**			
Cl_{ih} (1/h)	581.99	797.49	723.46
B_{max} (ng/ml)	11.03	11.03	–
K_d (ng/ml)	1.50	1.60	–
K_{abs} (1/h)	4.50	–	4.82
$LibT$ (min)	341.4	–	382.98
r_{chr}	0.12	0.12	0.12
**Patient 15**			
Cl_{ih} (1/h)	933.37	1403.98	908.13
B_{max} (ng/ml)	11.03	10.88	–
K_d (ng/ml)	1.51	1.42	–
K_{abs} (1/h)	4.50	–	4.87
$LibT$ (min)	59.0	–	165.7
r_{chr}	0.27	0.25	0.25
**Patient 20**			
Cl_{ih} (1/h)	594.58	566.70	530.74
B_{max} (ng/ml)	11.17	11.71	–
K_d (ng/ml)	1.47	1.02	–
K_{abs} (1/h)	4.50	–	2.09
$LibT$ (min)	393.3	–	154.9
r_{chr}	0.08	0.09	0.09
**Accuracy**	**NCA**	**Bayes**	**WSE**
**Patient 5**			
AUC_{24} (ng/ml · h)	106.1	105.1	107.6
$RMSE_c$	–	0.057	0.054
**Patient 15**			
AUC_{24} (ng/ml · h)	282.3	285.8	257.0
$RMSE_c$	–	0.177	0.074
**Patient 20**			
AUC_{24} (ng/ml · h)	155.4	152.6	156.4
$RMSE_c$	–	0.108	0.072

† Parameters with "–" value were not modified by the adaptive method.

PBPK model: Adaptive procedure 2 (Advagraf data).

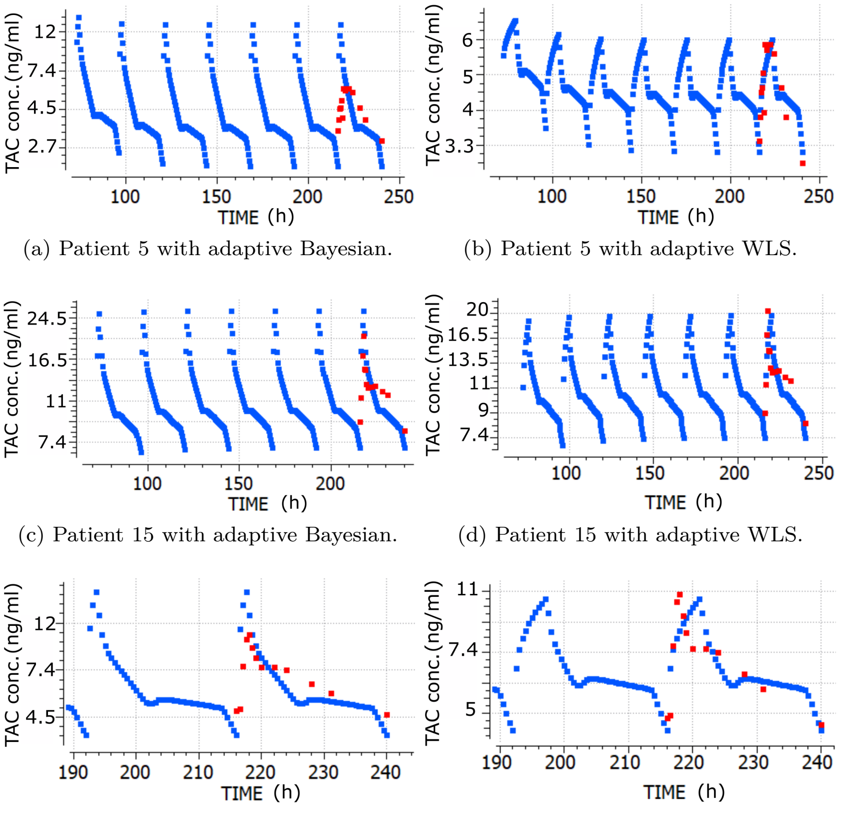

(a) Patient 5 with adaptive Bayesian. (b) Patient 5 with adaptive WLS.

(c) Patient 15 with adaptive Bayesian. (d) Patient 15 with adaptive WLS.

(e) Patient 20 with adaptive Bayesian. (f) Patient 20 with adaptive WLS.

Figure 16: Adapted PBPK model blood TAC concentrations (blue) (procedure 2) versus blood TAC measurements at day 8 (red) (log-transformed) for randomly selected patients. Time in hours.

Figure 16 shows the TAC concentration predictions for days 2 to 8 of the PBPK model adjusted with Bayesian and WLS adaptive methods (procedure 2), for randomly selected patients. Plots are centred on days 7 to 8 for Patient 20 to facilitate the inspection of TAC predictions versus measurements. The plots suggest that model TAC concentration predictions are more accurate with the WLS than the Bayesian adaptive method in Patients 5 and 20.

Table 9 shows the model parameters at the Prograf fitting and after the adjustment of the PBPK model with adaptive Bayesian and WLS methods. Patients 5 and 20 showed increased *LibT* from 100 min to 437.4 min and from 100 min to 319.5 min, unlike the smaller increase in Patient 15 (100 min to 222.5 min). The value of the *LibT* parameter cannot be adjusted by the Bayesian

method because it was not defined as a population parameter, which explains the poor performance of the Bayesian method compared with the WLS method.

Table 9: PBPK model parameters adjusted according to adaptive procedure 2 for randomly selected patients. Accuracy metrics: AUC_{24} and $RMSE_c$ (log-transformed).

**Params**†	**Prograf**	**Bayes**	**WSE**
**Patient 5**			
Cl_{ih} (1/h)	581.99	1702.0	1015.6
B_{max} (ng/ml)	11.03	10.4	–
K_d (ng/ml)	1.50	1.30	–
K_{abs} (1/h)	4.50	–	7.98
$LibT$ (min)	100	–	437.4
r_{chr}	0.12	0.11	0.11
**Patient 15**			
Cl_{ih} (1/h)	933.4	928.7	909.4
B_{max} (ng/ml)	11.03	11.0	–
K_d (ng/ml)	1.51	1.44	–
K_{abs} (1/h)	4.50	–	9.46
$LibT$ (min)	100	–	222.5
r_{chr}	0.27	0.25	0.25
**Patient 20**			
Cl_{ih} (1/h)	594.6	1384.5	1263.6
B_{max} (ng/ml)	11.2	10.8	–
K_d (ng/ml)	1.47	1.41	–
K_{abs} (1/h)	4.50	–	11.84
$LibT$ (min)	100	–	319.5
r_{chr}	0.08	0.09	0.08
**Accuracy**	**NCA**	**Bayes**	**WSE**
**Patient 5**			
AUC_{24} (ng/ml · h)	106.1	110.5	109.3
$RMSE_c$	–	0.389	0.073
**Patient 15**			
AUC_{24} (ng/ml · h)	282.3	274.2	274.6
$RMSE_c$	–	0.092	0.075
**Patient 20**			
AUC_{24} (ng/ml · h)	155.4	156.9	162.8
$RMSE_c$	–	0.196	0.085

† Parameters with "–" value were not modified by the adaptive method.

The changes in model parameters confirm the same trend detected in procedure 1 for these patients. The parameter r_{chr} was removed from the final adaptive evaluation because their variation was kept near zero for both methods.

PK model: Adaptive procedure 2 (Advagraf data).

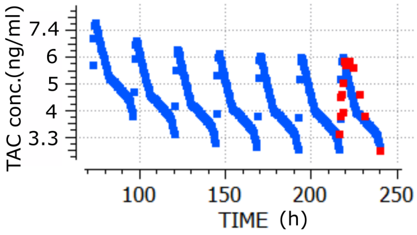

(a) Patient 5 with adaptive Bayesian.

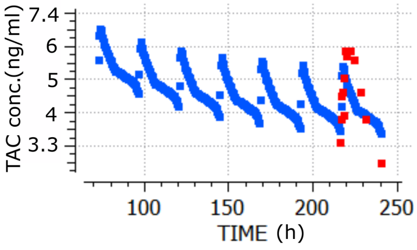

(b) Patient 5 with adaptive WLS.

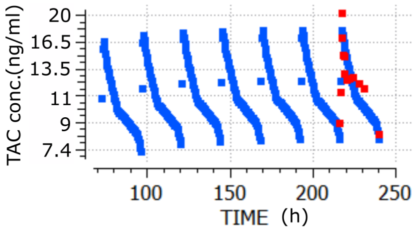

(c) Patient 15 with adaptive Bayesian.

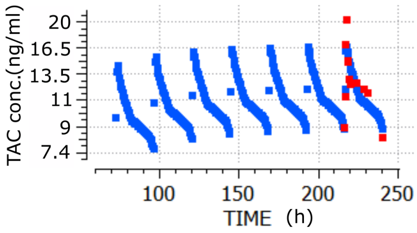

(d) Patient 15 adaptive WLS.

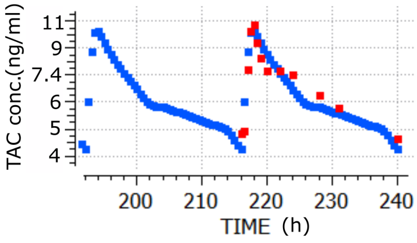

(e) Patient 20 with adaptive Bayesian.

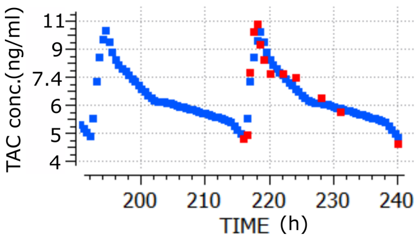

(f) Patient 20 with adaptive WLS.

Figure 17: Adapted PK model TAC concentrations (blue) (procedure2) versus TAC measurements at day 8 (red) (log-transformed) for randomly selected patients. Time in hours.

Figure 17 shows the TAC predictions for days 2 to 8 of the PK model adjusted with Bayesian and WLS adaptive methods (procedure 2) for randomly selected patients. The plots have been centred on days 7 to 8 for Patient 20 to facilitate the inspection of TAC concentrations. The plots suggest that model TAC concentration predictions have similar accuracy for the 2 adaptive techniques. This result agrees with the low correlation between FDT and the release rate of the TAC formulation (see main text).

Model parameter values at the Prograf fitting and after the adjustment of the PK model with adaptive Bayesian and WLS methods are shown in Table 10. The addition of FDT to the WLS adaptive method explains the parameter

Table 10: PK model parameters adapted to measurements on day 8 (procedure 2) for Patients 5, 15 and 20. Accuracy metrics: AUC_{24} and $RMSE_c$ (log-transformed).

**Params**	**Prograf**	**Bayes**	**WLS**
**Patient 5**			
Cl (l/h)	4.25	3.29	1.62
K_a (ml/h)	8.79	1.81	1.2
MTT (h)	0.66	0.90	0.62
r_{chr} (-)	0.28	0.28	0.28
FDT (h)	0.083	–	0.196
**Patient 15**			
Cl (l/h)	4.47	3.38	2.49
K_a (ml/h)	7.57	4.20	3.40
MTT (h)	0.56	0.62	0.55
r_{chr} (-)	0.29	0.29	0.29
FDT (h)	0.083	–	0.195
**Patient 20**			
Cl (l/h)	4.95	3.92	3.05
K_a (ml/h)	13.52	3.76	8.74
MTT (h)	0.68	0.83	0.27
r_{chr} (-)	0.28	0.28	0.27
FDT (h)	0.083	–	2.16
**Metrics**	**NCA**	**Bayes**	**WSE**
**Patient 5**			
AUC_{24} (ng/ml · h)	106.1	100.3	101.4
$RMSE_c$	–	0.122	0.111
**Patient 15**			
AUC_{24} (ng/ml · h)	282.3	273.4	278.0
$RMSE_c$	–	0.047	0.036
**Patient 20**			
AUC_{24} (ng/ml · h)	155.4	152.3	155.7
$RMSE_c$	–	0.071	0.061

differences between the adaptive techniques. In agreement with the PBPK model, $RMSE_c$ values indicate that the accuracy of the WLS-adapted PK model was better than the accuracy of the Bayesian-adapted PK model

Discussion

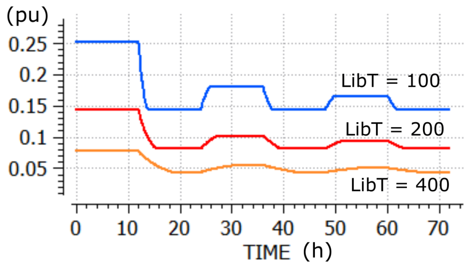

Figure 18: TAC bioavailability (F), calculated with the PBPK model as a function of $LibT$: blue ($LibT = 100$ min), red ($LibT = 200$ min) and orange ($LibT = 400$ min).

Bioavailability as a function of LibT. Figure 18 shows $F(t)$ as a function of $LibT$, as calculated by the PBPK model starting from a washout condition. After the initial transitory F reaches a near stable value with a temporal average equal to 15% ($LibT = 100$ min), 9% ($LibT = 200$ min) and 4% ($LibT = 400$ min). This reduction in F is associated with prolonged drug release. However, understanding this relationship requires further research.

Appendix: Covariates data

Only one covariate (BSA) was accepted by our PK TAC model, despite an intuitive tendency to include BW and age in a paediatric population. However, BSA is strongly correlated with both the patients' BW and age. The relationship between BSA and BW is included in the well-known Du Bois formula. Figure 19 shows the association between BSA and age in our population.

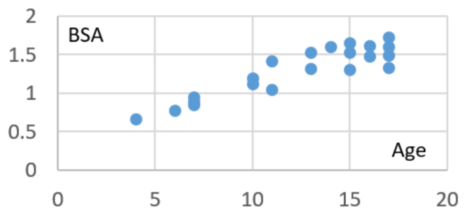

Figure 19: BSA (m^2) vs. age (years) in our paediatric population.

The BW–BSA association in our population is presented in Figure 20.

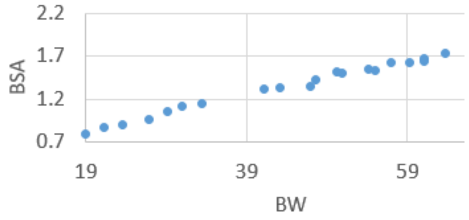

Figure 20: Relationship between BW (kg) - BSA (m^2) in our paediatric population.

Plots of figures 21, 22 and 23 show the covariate candidates obtained during the exploration phase.

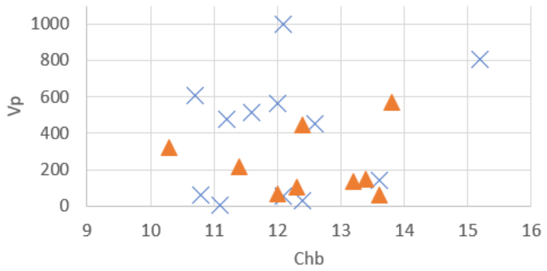

Figure 21: Relationship between Chb (g/dl) and gross estimated V_p (l) from exploratory study. Crosses indicate male patients and triangles indicate female patients.

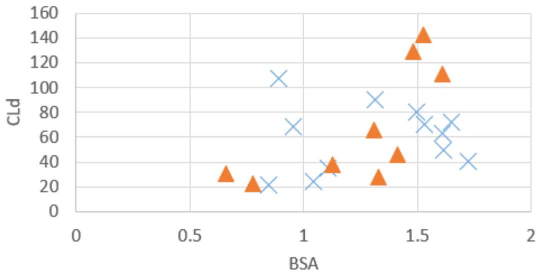

Figure 22: Relationship between BSA (m^2) and gross estimated CL_d (l/h) from the exploratory study. Crosses indicate male patients, and triangles indicate female patients.

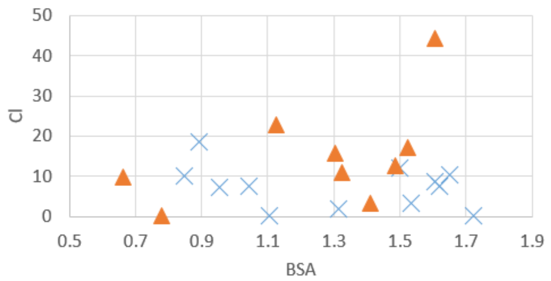

Figure 23: Relationship between BSA (m^2) and gross estimated CL (l/h) from the exploratory study. Crosses indicate male patients, and triangles indicate female patients.

References

Franc Andreu, Helena Colom, Josep M. Grinyó, Joan Torras, Josep M. Cruzado, and Nuria Lloberas. Development of a population pk model of tacrolimus for adaptive dosage control in stable kidney transplant patients. *Therapeutic Drug Monitoring*, 37:246–255, 2015.

J. M. Barbarino, C. E. Staatz, R. Venkataramanan, T. E. Klein, and R. B. Altman. Pharmgkb summary: cyclosporine and tacrolimus pathways. *Pharmacogenet Genomics*, 23(10):563–85, 2013.

Robert A Copeland. *Enzymes: A Practical Introduction to Structure, Mechanism, and Data Analysis.* Wiley-VCH, Inc., second edi edition, 2000. ISBN 0471359297.

Paul Deurenberg, Jan A. Weststrate, and Jaap C. Seidell. Body mass index as a measure of body fatness: age- and sex-specific prediction formulas. *British Journal of Nutrition*, 65(02), 2007.

DrugBank. Drugbank - tacrolimus (https://www.drugbank.ca/drugs/db00864), 2018.

M. Gertz, A. Harrison, J. B. Houston, and A. Galetin. Prediction of human intestinal first-pass metabolism of 25 cyp3a substrates from in vitro clearance and permeability data. *Drug Metab Dispos*, 38(7):1147–58, 2010.

Cécile Gérard, Jeanick Stocco, Anne Hulin, Benoit Blanchet, Céline Verstuyft, François Durand, Filomena Conti, Christophe Duvoux, and Michel Tod. Determination of the most influential sources of variability in tacrolimus trough blood concentrations in adult liver transplant recipients: a bottom-up approach. *The AAPS journal*, 16:379–91, 2014.

ILSI. Physiological parameter values for pbpk models. Report, International Life Sciences Institute, 1994.

William J. Jusko, Wojciech Piekoszewski, Goran B. Klintmalm, Mark S. Shaefer, Mary F. Hebert, Antoni A. Piergies, Charles C. Lee, Paul Schechter, and Qais A. Mekki. Pharmacokinetics of tacrolimus in liver transplant patients. *Clinical Pharmacology & Therapeutics*, 57(3):281–290, 1995.

A. W. Kelman, B. Whiting, and S. M. Bryson. Parameter optimisation in clinical pharmacokinetics. *Computer Programs in Biomedicine*, 14:239–248, 1982.

Toshikiro Kimura and Kazutaka Higaki. Gastrointestinal transit and drug absorption. *Biological and Pharmaceutical Bulletin*, 25(2):149–164, 2002.

Kazuko Nagase, Kazuhide Iwasaki, Katsuko Nozaki, and Kohsei Noda. Distribution and protein binding of fk506, a potent immunosuppressive macrolide lactone, in human blood and its uptake by erythrocytes. *Journal of Pharmacy and Pharmacology*, 46(2):113–117, 1994.

Michael Pelekis, Patrick Poulin, and Kannan Krishnan. An approach for incorporating tissue composition data into physiologically based pharmacokinetic models. *Toxicology and Industrial Health*, 11(5):511–522, 1995.

Patrick Poulin and Frank Peter Theil. A priori prediction of tissue: Plasma partition coefficients of drugs to facilitate the use of physiologically-based pharmacokinetic models in drug discovery. *Journal of Pharmaceutical Sciences*, 89:16–35, 2000.

Manuel Prado-Velasco. New approach based on mathematical modelling to compute drug bioavailability using the physpk™ biosimulation system. In *Conference: PAGE. Abstracts of the Annual Meeting of the Population Approach Group in Europe*. PAGE, 2018.

Karine Price, Sami Haddad, and Kannan Krishnan. Physiological modeling of age-specific changes in the pharmacokinetics of organic chemicals in children. *Journal of Toxicology and Environmental Health, Part A*, 66(5):417–433, 2003.

C. Schiller, C. P. Frohlich, T. Giessmann, W. Siegmund, H. Monnikes, N. Hosten, and W. Weitschies. Intestinal fluid volumes and transit of dosage forms as assessed by magnetic resonance imaging. *Aliment Pharmacol Ther*, 22(10):971–9, 2005.

T. H. Shin, M. J. Ho, S. R. Kim, S. H. Im, C. H. Kim, S. Lee, M. J. Kang, and Y. W. Choi. Formulation and in vivo pharmacokinetic evaluation of ethyl cellulose-coated sustained release multiple-unit system of tacrolimus. *Int J Biol Macromol*, 109:544–550, 2018.

P. L. Toutain and a. Bousquet-Melou. Free drug fraction vs. free drug concentration: A matter of frequent confusion. *Journal of Veterinary Pharmacology and Therapeutics*, 25:460–463, 2002.

C. Tron, M. Allard, A. Petitcollin, M. J. Ferrand-Sorre, M. C. Verdier, J. Querzerho-Raguideau, B. Blanchet, J. Le Priol, M. Roussel, Y. Deugnier, E. Bellissant, and F. Lemaitre. Tacrolimus diffusion across the peripheral mononuclear blood cell membrane: impact of drug transporters. *Fundam Clin Pharmacol*, 33(1):113–121, 2019.

Piekoszewski W, Chow FS, and Jusko WJ. Disposition of tacrolimus (fk 506) in rabbits: Role of red blood cell binding in hepatic clearance. *Drug Metab Dispos*, 21(4):690–698, 1993.

D. Worrall, K. Smith-Whitley, and L. Wells. Hemoglobin to hematocrit ratio: The strongest predictor of femoral head osteonecrosis in children with sickle cell disease. *J Pediatr Orthop*, 36(2):139–44, 2016.

H. Zahir, R. A. Nand, K. F. Brown, B. N. Tattam, and A. J. McLachlan. Validation of methods to study the distribution and protein binding of tacrolimus in human blood. *Journal of Pharmacological and Toxicological Methods*, 46 (1):27–35, 2001.
